# Evaluating genotyping‐in‐thousands by sequencing as a genetic monitoring tool for a climate sentinel mammal using non‐invasive and archival samples

**DOI:** 10.1002/ece3.10934

**Published:** 2024-02-07

**Authors:** Kate E. Arpin, Danielle A. Schmidt, Bryson M. F. Sjodin, Anthony L. Einfeldt, Kurt Galbreath, Michael A. Russello

**Affiliations:** ^1^ Department of Biology The University of British Columbia Kelowna British Columbia Canada; ^2^ Parks Canada East Kootenay G British Columbia Canada; ^3^ Department of Biology Northern Michigan University Marquette Michigan USA

**Keywords:** adaptive genetic variation, historical DNA analysis, indicator species, multiplexed targeted amplicon sequencing, non‐invasive genetic sampling, wildlife monitoring

## Abstract

Genetic tools for wildlife monitoring can provide valuable information on spatiotemporal population trends and connectivity, particularly in systems experiencing rapid environmental change. Multiplexed targeted amplicon sequencing techniques, such as genotyping‐in‐thousands by sequencing (GT‐seq), can provide cost‐effective approaches for collecting genetic data from low‐quality and quantity DNA samples, making them potentially useful for long‐term wildlife monitoring using non‐invasive and archival samples. Here, we developed a GT‐seq panel as a potential monitoring tool for the American pika (*Ochotona princeps*) and evaluated its performance when applied to traditional, non‐invasive, and archival samples, respectively. Specifically, we optimized a GT‐seq panel (307 single nucleotide polymorphisms (SNPs)) that included neutral, sex‐associated, and putatively adaptive SNPs using contemporary tissue samples (*n* = 77) from the Northern Rocky Mountains lineage of American pikas. The panel demonstrated high genotyping success (94.7%), low genotyping error (0.001%), and excellent performance identifying individuals, sex, relatedness, and population structure. We subsequently applied the GT‐seq panel to archival tissue (*n* = 17) and contemporary fecal pellet samples (*n* = 129) collected within the Canadian Rocky Mountains to evaluate its effectiveness. Although the panel demonstrated high efficacy with archival tissue samples (90.5% genotyping success, 0.0% genotyping error), this was not the case for the fecal pellet samples (79.7% genotyping success, 28.4% genotyping error) likely due to the exceptionally low quality/quantity of recovered DNA using the approaches implemented. Overall, our study reinforced GT‐seq as an effective tool using contemporary and archival tissue samples, providing future opportunities for temporal applications using historical specimens. Our results further highlight the need for additional optimization of sample and genetic data collection techniques prior to broader‐scale implementation of a non‐invasive genetic monitoring tool for American pikas.

## Graphical abstract

Genetic tools for wildlife monitoring can provide valuable information on spatiotemporal population trends and connectivity, particularly in systems experiencing rapid environmental change. Here, we developed a genotyping‐in‐thousands by sequencing (GT‐seq) panel (307 SNPs) as a potential monitoring tool for the American pika (*Ochotona princeps*) and evaluated its performance when applied to traditional, non‐invasive, and archival samples. The GT‐seq panel demonstrated high genotyping success, low genotyping error, and excellent performance identifying individuals, sex, relatedness, and population structure from contemporary and archival tissue samples, but this was not the case for the fecal pellet samples suggesting additional optimization of sample and genetic data collection techniques are required.

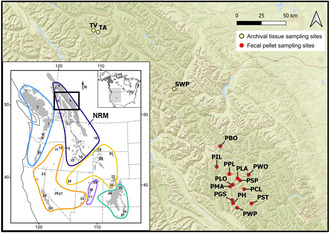

## 1 INTRODUCTION

Genetic information can provide valuable insights to inform wildlife conservation, particularly in the face of a changing climate (reviewed in Hohenlohe et al., 2021). Reconstructing patterns of genetic diversity, gene flow, and adaptive genetic variation over both space and time is fundamental for informing effective management strategies of both wildlife species and the ecosystems they inhabit (Bradburd & Ralph, 2019; Jensen & Leigh, 2022). This is exemplified by numerous spatiotemporal genetic studies yielding valuable conservation insights, particularly those studying endemic (Bi et al., 2019; Hawkins et al., 2016; Rubidge et al., 2012), endangered (Ford et al., 2020; Hartmann et al., 2014; van der Valk et al., 2019), and indicator species (Klingler et al., 2023).

Conservation genetic studies often rely on DNA from non‐invasive (e.g. hair and feces) and archival (e.g. skins, skeletons, or fluid‐preserved specimens in natural history museum collections) samples that have historically presented challenges for sequencing success and genotyping accuracy due to its low quantity/quality (Andrews et al., 2021; Raxworthy & Smith, 2021) and presence of PCR inhibitors (Kemp et al., 2020; Monteiro et al., 1997). Additionally, there is often an abundance of exogenous DNA within non‐invasive and archival samples, especially from microbial sources, which may cause off‐target co‐amplification during PCR that obscures the targeted signal (Carpenter et al., 2013; Perry et al., 2010; Taberlet et al., 1999). Together, these challenges may result in high genotyping error due to allelic dropout or false alleles (Smith & Wang, 2014; Taberlet et al., 1999), which can have detrimental effects on downstream applications such as individual identification or estimates of genetic diversity (Smith & Wang, 2014; Wang, 2018).

Multiplexed targeted amplicon sequencing (MTAS) approaches have recently been demonstrated to have high efficacy for genotyping single nucleotide polymorphisms (SNPs) from low‐quality DNA samples (Burgess et al., 2022; Eriksson et al., 2020; Hayward et al., 2022; Natesh et al., 2019; Schmidt, Campbell, et al., 2020). Such approaches can combine differentially informative SNPs, allowing researchers to simultaneously investigate patterns of genetic variation, sex‐biased dispersal, relatedness, and among‐site connectivity (Burgess et al., 2022; Hayward et al., 2022; Schmidt, Govindarajulu, et al., 2020). As consistent loci are targeted with each application, MTAS approaches also facilitate the collection of comparable data across multiple studies, which may be particularly useful for long‐term genetic monitoring projects (Chang et al., 2021, 2022; Hayward et al., 2022). Genotyping‐in‐thousands by sequencing (GT‐seq) is one MTAS approach that has gained recent popularity for its user‐friendly and cost‐effective workflow, which is capable of simultaneously genotyping hundreds of SNPs in thousands of samples (Campbell et al., 2015). GT‐seq amplifies and tags selected loci and individuals with unique barcodes, allowing all amplicons for all individuals to be combined together for sequencing prior to demultiplexing sequences bioinformatically (Campbell et al., 2015). Recent applications using DNA from field‐collected fecal and hair samples (Sitka black‐tailed deer, Burgess et al., 2022; polar bears, Hayward et al., 2022), as well as archival fish scales (Setzke et al., 2021), highlight the promise of GT‐seq as a spatiotemporal wildlife monitoring tool in study systems of high conservation or management priority.

Alpine ecosystems are expected to be disproportionately affected by climate change due to accelerated rates of warming at higher elevations (Dirnböck et al., 2011; Mountain Research Initiative EDW Working Group, 2015; Palomo, 2017), which may lead to changes in snowmelt runoff and altered freshwater supply (Kohler et al., 2010; Stewart et al., 2004). Yet, predicting the impact of environmental change on alpine ecosystems can be challenging due to complex, variable, and interacting climatic and biological factors (Chen et al., 2011; Lenoir & Svenning, 2015; Salzmann et al., 2014). Consequently, fine‐scale monitoring efforts may be needed to develop an accurate understanding of local ecological change. Population monitoring of indicator species, whose status is expected to reflect the conditions of their habitat, can provide direct insights into real‐time biological responses and trends to inform conservation and management priorities (Burger, 2006; Siddig et al., 2016).

**FIGURE 1:**
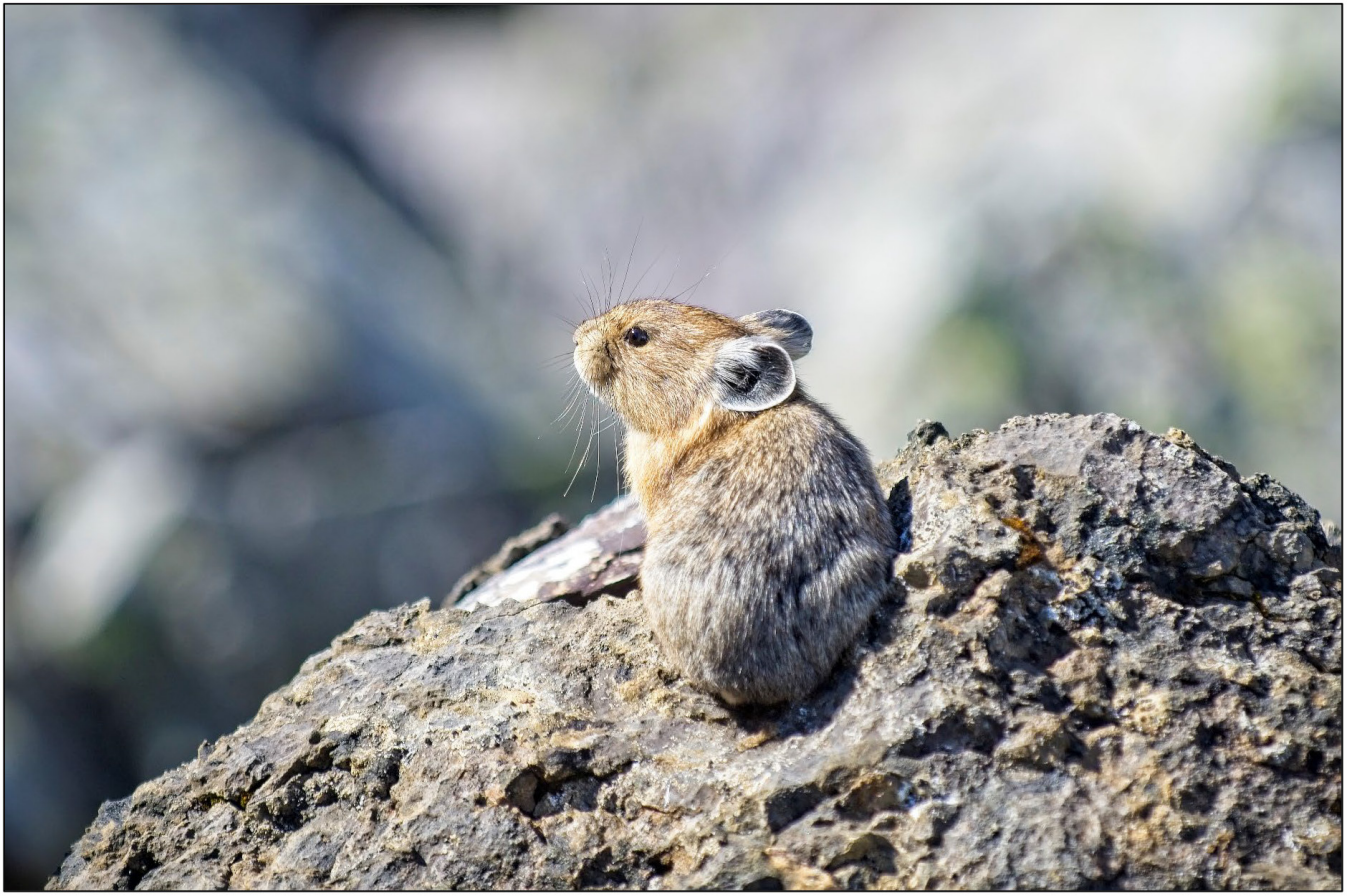
An American pika (*Ochotona princeps*) in Yoho National Park (Photocredit: Anthony Einfeldt).

The Canadian Rocky Mountains, which include Banff, Yoho, Kootenay, and Jasper National Parks (Alberta and British Columbia, Canada), encompass ecologically and culturally valuable alpine environments of particular management interest (UNESCO World Heritage Centre, 2023). An important mammalian indicator species in this region is the American pika (*Ochotona princeps*), a small lagomorph inhabiting high‐elevation alpine talus slopes across major mountain ranges in western North America (Figure 1; Beever et al., 2003; Smith & Weston, 1990; Wilkening & Ray, 2016). American pikas are acutely sensitive to environmental conditions as their lethal temperature is only ~3°C warmer than their basal body temperature; as a result, warming temperatures without sufficient compensation from behavioral thermoregulation can have fatal consequences (MacArthur & Wang, 1974; Smith, 1974). Accordingly, population declines in the American pika have been associated with temperature (Beever et al., 2003, 2010, 2011; Henry et al., 2012; Moritz et al., 2008; Stewart et al., 2015; Wilkening et al., 2011), as well as precipitation (Erb et al., 2011; Henry et al., 2012), elevation (Schmidt et al., 2021; Waterhouse et al., 2018) and available alpine talus habitat (Stewart et al., 2015). Within the Canadian Rocky Mountains, site occupancy of American pikas has been monitored annually for the last decade in Banff, Kootenay, and Yoho National Parks (conducted by Parks Canada; data available at www.open.canada.ca); however, patterns of connectivity across habitat patches, adaptive capacity of populations, and factors underlying demographic changes at some sites remain unknown. The availability of a genotyping tool—particularly one that is effective with non‐invasively sampled DNA sources—would improve the feasibility and cost‐effectiveness of long‐term monitoring efforts while minimizing disturbance to the system.

Here, we developed a GT‐seq panel for American pikas capable of identifying individuals and sex, classifying relatedness, reconstructing population connectivity, and quantifying putatively adaptive genetic variation at environmentally‐associated loci. We optimized the panel using contemporary tissue samples and applied it to non‐invasive fecal pellet samples collected at 13 monitored sites within the Canadian Rocky Mountains, as well as archival skin tissue samples collected in 1930 and 1945, to evaluate its efficacy as a long‐term monitoring tool for spatiotemporal applications.

## 2 MATERIALS AND METHODS

### 2.1 Study area and sample collection

**FIGURE 2:**
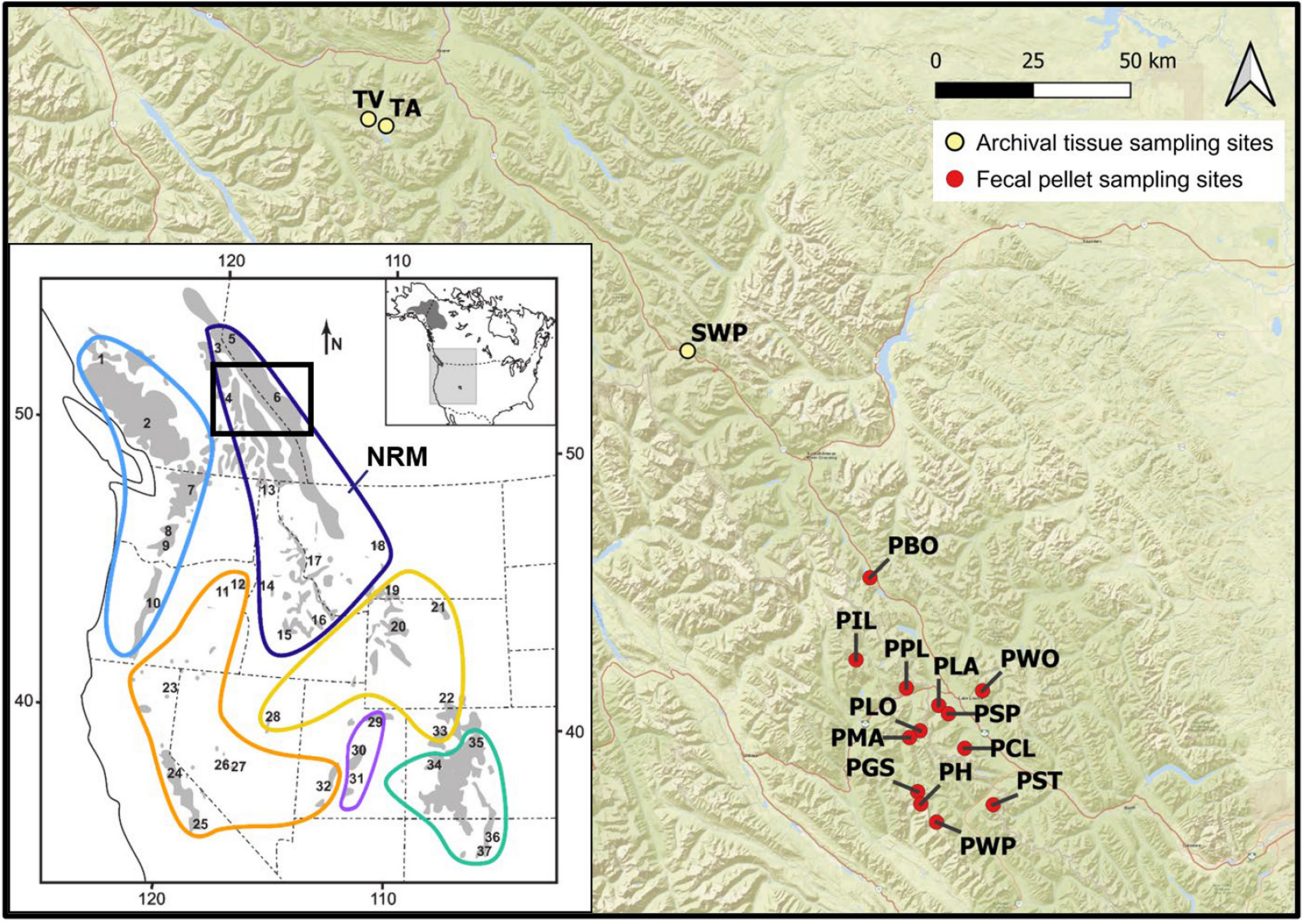
Locations of occupancy survey sites where fecal pellets were collected in 2021 and 2022 (red) and sites of archival specimen collection (yellow) within the Canadian Rocky Mountain National Parks (created in QGIS® using data provided by © Esri). The inset map (modified from Galbreath et al. (2009) and Schmidt et al. (in press)) shows contemporary tissue sample site collection (numbered sites) within the genomic lineages (circled in color) and American pika range (shaded gray). Notably, the Northern Rocky Mountain (NRM) lineage was recently revised by Schmidt et al. (in press) from the original Galbreath et al. (2009) to only include the northernmost cluster bordered in dark blue. The approximate sampling area of this present study is indicated by the black box within the NRM lineage.

We designed the American pika GT‐seq panel for primary application to monitored sites within the Canadian Rocky Mountains to supplement annual site occupancy surveys (Figure 2). Survey sites ranged from 3 to 65 km apart and were located within patches of alpine talus (~1800 to 2400 m above sea level) separated, in some cases, by natural features including lakes, forests, and valleys. Fecal pellet samples were collected from 13 sites in Banff, Kootenay, and Yoho National Parks from August–October 2021 (*n* = 59) and 2022 (*n* = 70) by Parks Canada staff and volunteers (Table 1; Figure 2). At each survey site, 3–12 pellets were collected per latrine depending on availability. All pellets at each latrine were individually collected with sterilized forceps (washed in a 10% bleach solution and air‐dried between latrines) and placed in a 2 mL tube containing 100% ethanol. Samples were shipped at ambient temperature and stored at 4°C storage until DNA extraction. Sampling efforts aimed to collect pellets that appeared to be recently deposited where possible (e.g. clumped together, moist, and soft when crushed), and up to six spatially distinct latrines were sampled per site (Table 1). For each sample, we combined up to 12 pellets from the same latrine to maximize DNA yield. As pikas are territorial, we assumed that pellets collected at each latrine belonged to a single individual, following previous studies (Castillo et al., 2014, 2016; Castillo & Epps, 2011; Klingler et al., 2023).

**TABLE 1:** Sample sizes (*n*) for fecal pellet samples collected among occupancy survey sites within Banff, Kootenay, and Yoho National Parks, British Columbia and Alberta, Canada in 2021 and 2022, as well as archival tissue samples collected among three sites in Jasper National Park in 1930 and 1945.

	Site ID	National Park	Site name	Source	Contemporary *n*		Archival *n*	
					2021	2022	1930	1945
Fecal pellets	PBO	Banff	Bow Summit	Parks Canada	5	6	–	–
	PCL	Banff	Consolation Lakes	Parks Canada	5	7	–	–
	PLA	Banff	Lake Agnes	Parks Canada	5	6	–	–
	PSP	Banff	Saddleback Pass	Parks Canada	5	6	–	–
	PWO	Banff	Wolverine Bowl	Parks Canada	3	6	–	–
	PGS	Kootenay	Goodsir	Parks Canada	5	5	–	–
	PH	Kootenay	Helmet	Parks Canada	5	4	–	–
	PST	Kootenay	Stanley Glacier	Parks Canada	5	6	–	–
	PWP	Kootenay	Wolverine Pass	Parks Canada	2	3	–	–
	PIL	Yoho	Iceline	Parks Canada	5	6	–	–
	PLO	Yoho	Lake Oesa	Parks Canada	5	6	–	–
	PMA	Yoho	McArthur	Parks Canada	5	6	–	–
	PPL	Yoho	Paget	Parks Canada	4	3	–	–
Archival tissue	SWP	Jasper	Sunwapta Pass	Canadian Museum of Nature	–	–	–	3
	TA	Jasper	Tonquin Valley	Cowan Vertebrate Museum	–	–	–	3
	TV	Jasper	Tonquin Valley	Canadian Museum of Nature	–	–	11	–

We also obtained archival samples to investigate the efficacy of the developed GT‐seq panel to successfully genotype historical tissue samples (Table 1; Table B1; Figure 2). We received skin clips (approximately 10 mm × 3 mm × 1 mm in size) from archival American pika specimens (*n* = 17) originally collected in 1930 and 1945 from three sites within Jasper National Park (Alberta, Canada) from the Cowan Vertebrate Museum (now merged with the Beaty Biodiversity Museum, Vancouver, British Columbia) and the Canadian Museum of Nature (Ottawa, Ontario; Table 1; Figure 2). Skin clips were stored in dry tubes at ambient temperatures prior to DNA extraction. Specimen accession numbers are listed in Table B1.

The high‐quality liver tissue samples used in GT‐seq panel optimization were previously acquired by Galbreath et al. (2009) and accessioned at the Cornell University Museum of Vertebrates (accession numbers and metadata provided in Table B1). After their collection in 2004 and 2005, these samples had been frozen and stored at −80°C prior to use in this project. We thawed approximately 15 mg of each sample for DNA extraction.

### 2.2 SNP selection for GT‐seq panel design

We selected neutral, sex‐associated, and putatively adaptive SNPs for inclusion in the GT‐seq panel through various methodologies and workflows. All datasets involved in SNP selection are listed and described in Table B2.

#### 2.2.1 Neutral SNPs

To maximize applicability to the Canadian Rocky Mountains, we used restriction site‐associated DNA sequencing (RADseq; Baird et al., 2008) data previously generated (Schmidt et al., in press) from 77 individuals across nine sites within the Northern Rocky Mountains (NRM) genetic lineage (samples originally collected by Galbreath et al., 2009; Figure 2). Though ten sites are geographically located within the NRM genetic lineage, we had excluded site 18 due to evidence of admixture among lineages (Schmidt et al., in press; Figure 2). To discover neutral SNPs, we first processed raw paired‐end reads with the *process_radtags* module of STACKS v. 2.3e (Rochette et al., 2019) to demultiplex, clean, and trim reads to 94 bp. We then aligned these data to the American pika reference genome OchPri4.0 (Sjodin et al., 2021) using BWA‐MEM v. 0.7.17 with default parameters (Li, 2013). Next, we identified and genotyped all SNPs among individuals with the *gstacks* module in STACKS v. 2.3e (Rochette et al., 2019) and then called SNPs that adhered to selected parameters using the STACKS *populations* module. We called SNPs that were present in at least 80% of individuals, had a minimum minor allele frequency of 0.03, and had a maximum observed heterozygosity of 0.5. A prior sensitivity analysis from Schmidt et al. (in press) indicated that these thresholds were optimal to maximize the number of loci retained while minimizing missing data. We removed individuals with >20% missing data and a depth of coverage <6× and re‐ran *populations* with the optimal parameters as stated above. We subsequently filtered loci for a minimum mean genotype depth of 10, maximum mean genotype depth of 100, and maximum of 20% missing data across all individuals using VCFTOOLS v. 0.1.16 (Danecek et al., 2011). Pairwise relatedness among remaining individuals was assessed using the R package *related* (Pew et al., 2015), and we removed one individual of each pair demonstrating relatedness values >0.9. We removed remaining SNPs located on unplaced scaffolds and the X chromosome to retain only autosomal chromosomes. Putative paralogs were identified using the R package *HDplot* as described in McKinney et al. (2017) and removed for downstream analyses. We also removed SNPs identified as having significant associations with the WorldClim 2.0 bioclimatic variables (Fick & Hijmans, 2017) using Latent Factor Mixed Modeling employed by the *lfmm_ridge* function in the R package *lfmm* (Caye et al., 2019; see Appendix A for methods) and redundancy analysis (RDA) employed by the R package *vegan* (Oksanen et al., 2016; Appendix A). Additional outlier SNPs were detected with R package *pcadapt* (Privé et al., 2020), and SNPs with *p*‐values <.05 after a Benjamini‐Hochberg correction were removed. Finally, we removed SNPs that deviated from Hardy–Weinberg equilibrium in over 50% of sampled sites using the script *filter_hwe_by_pop.pl* (https://github.com/jpuritz/dDocent/blob/master/scripts/filter_hwe_by_pop.pl).

We filtered the retained neutral SNPs (*n* = 20,993) for physical linkage by removing loci within a 20 kb window of each other (Carneiro et al., 2011) using VCFTOOLS; of the resulting SNPs (*n* = 15,480), we retained only those between the 40th—60th base pair position on the contig to facilitate primer design (*n* = 3071 SNPs). Of these, we selected a random subset of 700 SNPs distributed across the genome. To investigate the effect of locus dropout during primer design and panel optimization on downstream panel applications, we selected a random subset of 350 SNPs from these 700 SNPs to conduct parallel analyses of individual identification, relatedness, and population structure relative to the 700 SNP and full 15,480 SNP datasets.

For the 350 and 700 SNP datasets, we calculated the multi‐locus probability of identity (*p*_ID_) and probability of identity between siblings (*p*_IDSIB_) using GenAlEx v. 6.503 (Peakall & Smouse, 2006; Smouse & Peakall, 2012) to evaluate the ability of each dataset to identify individuals. We then assessed the efficacy of each of the three datasets (350 SNP, 700 SNP, full 15,480 SNP) to estimate relatedness. We used the *sim_rel* function of the R package *irelr* (Gonçalves da Silva & Russello, 2011) to simulate 1,000,000 dyads each of unrelated, full‐siblings (FS), and half‐siblings (HS) relationships, and then compared the probability of misclassification following the approach described in the *use‐irelr* vignette (https://github.com/andersgs/irelr/). We also compared population genetic parameters by site and globally (i.e. among all sites), including the proportion of missing data, mean allelic richness, mean minor allele frequency, observed heterozygosity, expected heterozygosity, and inbreeding coefficient (*F*_IS_) using the R packages *adegenet* (Jombart, 2008) and *hierfstat* (Goudet, 2005). We then assessed population structure using the maximum likelihood approach employed by ADMIXTURE v. 1.3.0 (Alexander et al., 2009), which estimates the proportion of genetic assignment of each individual to a given number (*K*) of genetic clusters. For this analysis, we ran 10 iterations for *K* values 1–11. The *K* value with the lowest mean cross‐validation (CV) error rate was determined to be the optimal *K*, as recommended by the developers. Finally, we conducted a principal component analysis (PCA) using the R package *smartsnp* (Herrando‐Pérez et al., 2021) and calculated pairwise estimates of population differentiation (*F*_ST_) using the R package *hierfstat* (Goudet, 2005) to compare reconstructed patterns of among‐site structure between the three datasets. The *boot.ppfst* function of *hierfstat* was used to calculate confidence intervals (0.95) using 1000 bootstraps, which provided an indication of significant deviations of pairwise *F*_ST_ from zero.

#### 2.2.2 Sex‐associated SNPs

To identify sex‐associated SNPs, we used previously generated RADseq data (Schmidt et al., in press) from a randomly selected subsample of 128 reference individuals of known phenotypic sex (*n* = 64 of each males and females) across all lineages range‐wide (Galbreath et al., 2009). Phenotypic sex had previously been classified through physical examination upon specimen collection. We aligned raw reads to the reference genome and processed them through the STACKS v. 2.3e workflow (Rochette et al., 2019) as previously described. We included requirements for at least 80% of individuals to have a specific locus to call a SNP, a minor allele frequency >0.05, and an observed heterozygosity <0.5. We used RDA to identify sex‐associated SNPs as loci that demonstrated a strong association with phenotypic sex (Carrier et al., 2020) as implemented in the R package *vegan* (Oksanen et al., 2016). Only loci over eight standard deviations from the mean SNP loading value were further explored (*n* = 14); this threshold was selected to retain a working number of SNPs for panel design.

To validate the performance of these putatively sex‐associated SNPs to diagnose phenotypic sex, we tested their application to samples across all combined lineages (i.e. range‐wide) and then from NRM only, which allowed us to directly demonstrate applicability to the Canadian Rocky Mountains sites. First, we genotyped a different set of 128 test individuals at the selected putatively sex‐associated SNPs (64 males and 64 females from across all lineages; Schmidt et al., in press). All test individuals were of known phenotypic sex, which had been previously identified from physical examination during specimen collection. We then conducted individual assignment and Bayesian clustering analyses on the test dataset relative to the reference dataset. We used the individual assignment approach of Rannala and Mountain (1997), as implemented in GENECLASS2 v. 2.0.h (Piry et al., 2004) to assign test individuals to defined phenotypic reference male and female groups. As a supplementary assignment analysis, we also used the Bayesian clustering analysis implemented in STRUCTURE v. 2.3.4 (Pritchard et al., 2000) using ten replicates at *K* = 2, with 500,000 Markov Chain Monte‐Carlo iterations after a burn‐in period of 100,000. Prior population information was included in the analysis to use the phenotypic sex assignments of the reference population as a baseline for assigning genotypic sex. Replicates were combined using CLUMPP v. 1.1.2 (Jakobsson & Rosenberg, 2007) and visualized using DISTRUCT v. 1.1 (Rosenberg, 2004). We then proceeded to validate sex‐associated SNPs for efficacy on a subset of samples from the NRM lineage (*n* = 26) using the previously described Bayesian clustering and individual assignment analyses. The samples included here were not previously used in SNP discovery. Retained sex‐associated SNPs were annotated with the Ensembl Variant Effect Predictor (VEP; McLaren et al., 2016), which used the American pika reference genome OchPri4.0 (Sjodin et al., 2021) to identify all genes within a 20,000 bp window of the SNP (Carneiro et al., 2011).

#### 2.2.3 Adaptive SNPs

We included putatively adaptive SNPs on the GT‐seq panel to enable future research to track the shifts in allele frequencies in association with changing environments. We selected putatively adaptive SNPs from three previously generated datasets: robust NRM outliers (i.e. NRM‐specific SNPs indicated as outliers by two different methods; see Section 2.2.1 and Appendix A), SNPs annotated to positively selected genes (Sjodin & Russello, 2022), and elevation‐associated variants (Schmidt et al., 2021). From the set of environmentally‐associated SNPs detected previously within the NRM lineage (see Section 2.2.1 and Appendix A), we first considered outliers identified within both RDA and LFMM analyses (i.e. robust NRM outliers) for inclusion in the panel (*n* = 155). We identified the genes annotated to these SNPs with the Ensembl VEP (McLaren et al., 2016) as described in Section 2.2.2 and investigated them in the literature for evidence of association with climate adaptation, using the search terms in Google Scholar: “[gene symbol]” OR “[full gene name]” “cold stress” OR “cold response” OR “cold resistance” OR “hypoxia” OR “high altitude” OR “UV damage” OR “climate” OR “heat stress” OR “precipitation” OR “water stress.” For each unique gene, we reviewed the top 20 search results; outlier SNPs associated with genes demonstrating a potential link to environmental adaptation were included in the initial panel. We also considered candidate SNPs from positively selected genes (PSGs) originally identified in a comparative genomics study using the reference genomes of American pika and eight other mammal species (Sjodin & Russello, 2022). Of these candidate SNPs, only those also independently classified as robust outliers within the NRM lineage or among all lineages (approach described in Appendix A) were included in the GT‐seq panel. Lastly, we considered climate‐associated outlier SNPs that were previously identified in a population genomic study of American pika populations sampled along elevational transects in Tweedsmuir South Provincial Park, BC, and North Cascades National Park (NOCA), Washington, USA (Schmidt et al., 2021). We investigated the correlation between allele frequency and elevation along the transects for all climate‐associated outlier SNPs detected in Schmidt et al. (2021) using both linear and logistic regression implemented in Microsoft Excel; SNPs demonstrating significant correlations (*p* < .05) using at least one method were retained. Of these SNPs, we removed loci with >20% missing data and a minor allele frequency <.03 in the NRM samples to support their applicability to the studied system.

### 2.3 Panel optimization using high‐quality tissue DNA

DNA sequences containing all neutral, sex‐associated, and putatively adaptive SNPs for the GT‐seq panel development were sent to GTseek LLC (https://gtseek.com/) for custom locus‐specific primer design. Prior to testing GT‐seq panel application to low‐quality sample types, we first conducted multiple rounds of optimization using high‐quality tissue samples. We constructed the initial GT‐seq library using American pika liver tissue samples (*n* = 77 + 8 replicates) collected by Galbreath et al. (2009) and accessioned at the Cornell University Museum of Vertebrates from nine sites across the NRM lineage (Figure 2). As these samples had been previously genotyped using RADseq (Schmidt et al., in press), selecting these samples for GT‐seq panel optimization enabled us to compare the genotyping concordance between RADseq and GT‐seq. Approximately 10% of samples were replicated (i.e. technical replicates) within each round of optimization to facilitate estimates of genotyping error.

First, we extracted DNA from 15 mg of liver tissue for each sample using a Qiagen DNeasy® Blood & Tissue Kit following the manufacturer's protocol with RNAse A treatment. We then quantified DNA extracts using a Qubit™ Flex Fluorometer with a 1× dsDNA High Sensitivity Assay Kit and manually standardized each sample to 15 ng/μL. GT‐seq library construction followed the procedure from Campbell et al. (2015) as modified in Burgess et al. (2022). PCR2 products were quantified using the Qubit™ Flex Fluorometer, and each sample was manually normalized to 20 ng/μL before pooling 5 μL of each normalized product. We then purified the pooled library using a Qiagen MinElute® PCR Purification Kit and eluted the product into 24 μL of ddH_2_O. Size fragment distribution was evaluated using a D1000 ScreenTape on an Agilent 2200 TapeStation prior to sequencing on an Illumina MiniSeq™.

Raw sequencing reads were demultiplexed by sample and genotyped (*Gtseq_Genotyper_v3.pl*) using the GT‐seq bioinformatics pipeline (https://github.com/GTseq; Campbell et al., 2015). For each round of GT‐seq panel optimization, we evaluated the prevalence of primer interactions, off‐target reads, and overamplification using *Gtseq_SeqTest.pl* and *Gtseq_Primer‐Interaction‐Test.pl* (https://github.com/GTseq) and removed all SNPs negatively impacting genotyping success from subsequent libraries. Specifically, we removed loci with >1% of all reads or primers accounting for >1% of all primer interactions, as well as one SNP of each double complement primer pair. Next, we removed samples and then SNPs with >50% missing data prior to evaluating genotyping concordance and error. Genotyping concordance was calculated between all GT‐seq genotypes and RADseq genotypes for unique samples using the script *genoerrorcalc.py* (https://github.com/bsjodin/genoerrorcalc). We investigated within GT‐seq genotyping error by comparing the frequency of genotype mismatches between replicated samples (*n* = 8) where neither replicate had missing data. After three rounds of optimization, we removed samples and then SNPs with >50% missing data to yield a final GT‐seq panel for further application.

### 2.4 Panel application to fecal pellet DNA

#### 2.4.1 Genetic data collection

Whole fecal pellets were stored in 100% ethanol at 4°C until DNA extraction. We extracted fecal DNA using a QIAamp® Fast DNA Stool Mini Kit as per the manufacturer's protocol, which included homogenizing samples in InhibitEX Buffer. We subsequently quantified samples using a Qubit™ Flex Fluorometer with a 1× dsDNA High Sensitivity Assay Kit. To help standardize reads per sample and maximize DNA retained, we normalized samples to 3 ng/μL DNA through either manual dilution with ddH_2_O or concentration using a SpeedVac Vacuum Concentrator. GT‐seq library construction for the fecal pellet samples (*n* = 129 + 14 replicates) followed the workflow used for the initial tissue DNA libraries, except that primers were divided into two separate pools to reduce the likelihood of primer interactions and maximize template DNA concentration following Burgess et al. (2022). We eluted pooled and purified libraries into 24 μL of ddH_2_O and evaluated size fragment distribution using a D1000 ScreenTape on an Agilent 2200 TapeStation prior to sequencing on an Illumina MiniSeq™.

As this first fecal DNA library yielded low genotyping success (see Results), we conducted several preliminary experiments to evaluate how different variables within the GT‐seq workflow affected fecal pellet genotype success and found that DNA fragment size‐selection showed a measurable improvement (data not shown). We therefore prepared a second fecal DNA library as described above with a supplementary size selection step to reduce off‐target amplification. For the size selection, we loaded pooled and purified PCR2 products into a Pippin Prep™ DNA Size Selection System using a 2% Agarose Gel Cassette with an internal control and processed samples according to the manufacturer's protocol. Given the expected fragment length range of our PCR2 products, we retained only those DNA fragments that were between 181 and 261 bp. We purified the size‐selected products using a Qiagen MinElute® PCR Purification Kit and eluted the library into 15 μL of ddH_2_O before sequencing on an Illumina MiniSeq™. We bioinformatically combined reads for each unique sample between primer pool replicates across both sequenced libraries and genotyped them using the GT‐seq pipeline as described above. To retain informative SNPs, we removed SNPs and then samples with >50% missing data, followed by SNPs with a minor allele frequency <0.03. We calculated genotyping error for all retained replicates within the filtered datasets.

#### 2.4.2 Evaluation as a monitoring tool

We evaluated the ability of our data to accurately identify individuals by calculating the multi‐locus probability of identity (*p*_ID_; Waits et al., 2001) and the probability of identity for siblings (*p*_IDSIBS_) using GenAlEx v. 6.503 (Peakall & Smouse, 2006; Smouse & Peakall, 2012). As the same latrines may have been sampled during both years, and pikas may have contributed to multiple latrines, assessing the ability of the GT‐seq panel to differentiate unique samples was important to identify and remove replicates from downstream analyses. To that end, we used the maximum likelihood pedigree reconstruction method implemented in COLONY v. 2.0.6.8 (Jones & Wang, 2010) to identify unique individuals by comparing genotypes given pre‐determined genotyping error rates for each locus (Wang, 2018). All default parameters were used with the following modifications: (1) assumed male and female polygamy; (2) allowed for clones; and (3) defined genotyping error. The genotyping error used in this analysis was calculated on a locus‐by‐locus basis based on values estimated between technical replicates. We secondarily identified replicates using the multi‐locus matches option in GenAlEx v. 6.503 (Peakall & Smouse, 2006; Smouse & Peakall, 2012), which flags samples with identical genotypes at all loci. Technical replicates were retained for the individual identification analyses using COLONY and GenAlEx (Multilocus Matches) to provide an internal control.

We compared the ability of our SNP data to classify pairwise relatedness between individuals using the *sim_rel* function within the R package *irelr* (Gonçalves da Silva & Russello, 2011). We simulated 1,000,000 dyads of FS, HSs, and unrelated (UR) individuals and estimated the probability of misclassification following the approach described in the *use‐irelr* vignette (https://github.com/andersgs/irelr/).

To evaluate among‐site patterns of genetic structure, we implemented the maximum likelihood approach by ADMIXTURE 1.3.0 (Alexander et al., 2009) to estimate the proportion of genetic assignment of each individual to *K* genetic clusters. We tested *K* from 1 to 15 (i.e. 1 to *n* + 2, where *n* is the number of a priori sites) using the recommended fivefold CV error method (Alexander et al., 2009) across ten replicates and selected the *K* with the lowest mean CV error. Replicated runs were combined into a consensus figure using CLUMPAK (Kopelman et al., 2015). We also reconstructed patterns of among‐site genetic variation with a PCA using the R package *smartsnp* (Herrando‐Pérez et al., 2021). We calculated pairwise and global genetic differentiation (*F*_ST_) among sites using the R package *hierfstat* (Goudet, 2005), and we used the *boot.ppfst* function to evaluate significant deviations of pairwise *F*_ST_ from zero by calculating confidence intervals (0.95) using 1000 bootstraps. Lastly, we conducted a Mantel test in GenAlEx v. 6.503 (Peakall & Smouse, 2006; Smouse & Peakall, 2012) over 9999 iterations to compare genetic and geographic distances among samples.

### 2.5 Panel application to archival tissue DNA

We processed archival samples in an isolated historical DNA laboratory (The University of British Columbia, Kelowna, British Columbia) prior to PCR1 to prevent contamination with modern DNA. We used a Qiagen DNeasy® Blood & Tissue Kit to extract archival DNA following the manufacturer's protocol and subsequently quantified samples using a Qubit™ Flex Fluorometer with a 1× dsDNA High Sensitivity Assay Kit. We prepared archival samples (*n* = 17 + 1 replicate) for sequencing using the same GT‐seq protocol as for fecal DNA, including the use of two primer pools, size‐selecting DNA fragments from pooled and purified PCR2 products using a Pippin Prep™, and sequencing purified products on an Illumina MiniSeq™. We genotyped samples and subsequently calculated genotyping error as previously described.

## 3 RESULTS

### 3.1 SNP selection for GT‐seq panel

*p*_ID_ and *p*_IDSIBS_ estimates were exceptionally low for both the 700 SNP (*p*_ID_: 1.9E−143; *p*_IDSIBS_: 8.0E−73) and 350 SNP (*p*_ID_: 5.7E−74; *p*_IDSIBS_: 9.3E−38) datasets (Table 2), respectively, requiring a minimum of 15 and 17 SNPs to attain *p*_ID_ < .001 and both requiring 29 SNPs to attain *p*_IDSIBS_ < .001. Misclassification rates among relatedness categories were low for each dataset (Table B3). Specifically, the 700 and 350 SNP datasets had a misclassification probability of 0.4% and 2.9% for full siblings, 1.4% and 7.5% for half siblings, and 1.0% and 4.7% for unrelated individuals, respectively (Table B3). The initial set of 700 putatively neutral SNPs and random 350 SNP subset yielded similar patterns of within‐site genetic variation (Table 2) and among‐site population structure (Table B4; Figures B1 and B2) among the nine NRM sites relative to the full 15,480 SNP dataset.

**TABLE 2:** Population genetic parameters and *p*_ID_ and *p*_IDSIBS_ estimates of neutral SNP datasets for GT‐seq panel SNP selection using *adegenet*, *hierfstat*, and GenAlEx v. 6.5, where allelic richness = *A*_R_, MAF = minor allele frequency, *H*_O_ = observed heterozygosity and *H*_E_ = expected heterozygosity.

	Site	*n*	Missing data (%)	*p*_ID_	*p*_IDSIBS_	*A*_R_	MAF	*H*_O_	*H*_E_	*F*_IS_	Global *F*_ST_
350 SNPs	3	7	2.08	4.77E−32	9.28E−17	1.1105	0.0761	0.1086	0.1107	0.0049	0.2811
	4	6	3.38	2.18E−39	1.54E−20	1.1379	0.0951	0.1457	0.1374	−0.0569	
	5	3	1.90	1.46E−20	6.38E−11	1.0785	0.0516	0.0755	0.0798	−0.0086	
	6	17	3.76	4.12E−57	1.7E−29	1.1897	0.1338	0.2012	0.1893	−0.0454	
	13	14	4.24	4.09E−57	1.44E−29	1.1903	0.1351	0.2083	0.1897	−0.0816	
	14	12	3.86	4.36E−61	1.63E−31	1.2046	0.1426	0.1933	0.2052	0.0625	
	15	7	0.69	7.92E−51	1.71E−26	1.1761	0.1256	0.1866	0.1752	−0.0613	
	16	7	0.98	5.86E−59	1.97E−30	1.2033	0.1376	0.2083	0.2029	−0.0334	
	17	4	1.79	1.25E−55	1.11E−28	1.2038	0.1292	0.1983	0.2048	0.0046	
Combined		77	2.98	5.74E‐74	9.27E−38	1.1661	0.1141	0.1695	0.1670	−0.0151	
700 SNPs	3	7	2.33	8.45E−69	1.73E−35	1.1196	0.0812	0.1230	0.1193	−0.0332	0.2819
	4	6	3.14	1.75E−74	1.49E−38	1.1316	0.0912	0.1394	0.1310	−0.0607	
	5	3	1.62	5.8E−38	8.88E−20	1.0731	0.0463	0.0692	0.0744	0.0089	
	6	17	3.55	1.5E−104	1.38E−53	1.1740	0.1210	0.1834	0.1737	−0.0371	
	13	14	4.02	2E−116	7.02E−60	1.1952	0.1382	0.2026	0.1949	−0.0357	
	14	12	3.46	9.3E−114	2.62E−58	1.1914	0.1322	0.1829	0.1918	0.0453	
	15	7	1.00	2.7E−101	2.57E−52	1.1765	0.1238	0.1811	0.1759	−0.0285	
	16	7	1.37	5.4E−113	3.85E−58	1.1965	0.1350	0.2000	0.1964	−0.0261	
	17	4	2.25	1.3E−110	1.19E−56	1.2042	0.1286	0.2005	0.2051	−0.0059	
Combined		77	2.91	1.9E−143	8.03E‐73	1.1625	0.1108	0.1647	0.1634	−0.0077	
15,480 SNPs	3	7	2.10	*NA*	*NA*	1.1151	0.0779	0.1191	0.1147	−0.0353	0.2830
	4	6	2.82			1.1329	0.0904	0.1446	0.1316	−0.0877	
	5	3	1.55			1.0885	0.0558	0.0875	0.0882	−0.0341	
	6	17	3.40			1.1751	0.1225	0.1795	0.1749	−0.0194	
	13	14	3.83			1.1920	0.1353	0.2018	0.1916	−0.0438	
	14	12	3.56			1.1947	0.1354	0.1838	0.1953	0.0652	
	15	7	1.09			1.1831	0.1271	0.1899	0.1826	−0.0406	
	16	7	1.29			1.1890	0.1296	0.1941	0.1885	−0.0343	
	17	4	2.40			1.1992	0.1268	0.1900	0.2009	0.0176	
*Combined*		77	2.81			1.1633	0.1112	0.1656	0.1640	−0.0097	

*Note*: *p*_ID_ and *p*_IDSIBS_ were not computed for the full 15,480 SNP dataset due to computational limitations.

**FIGURE 3:**
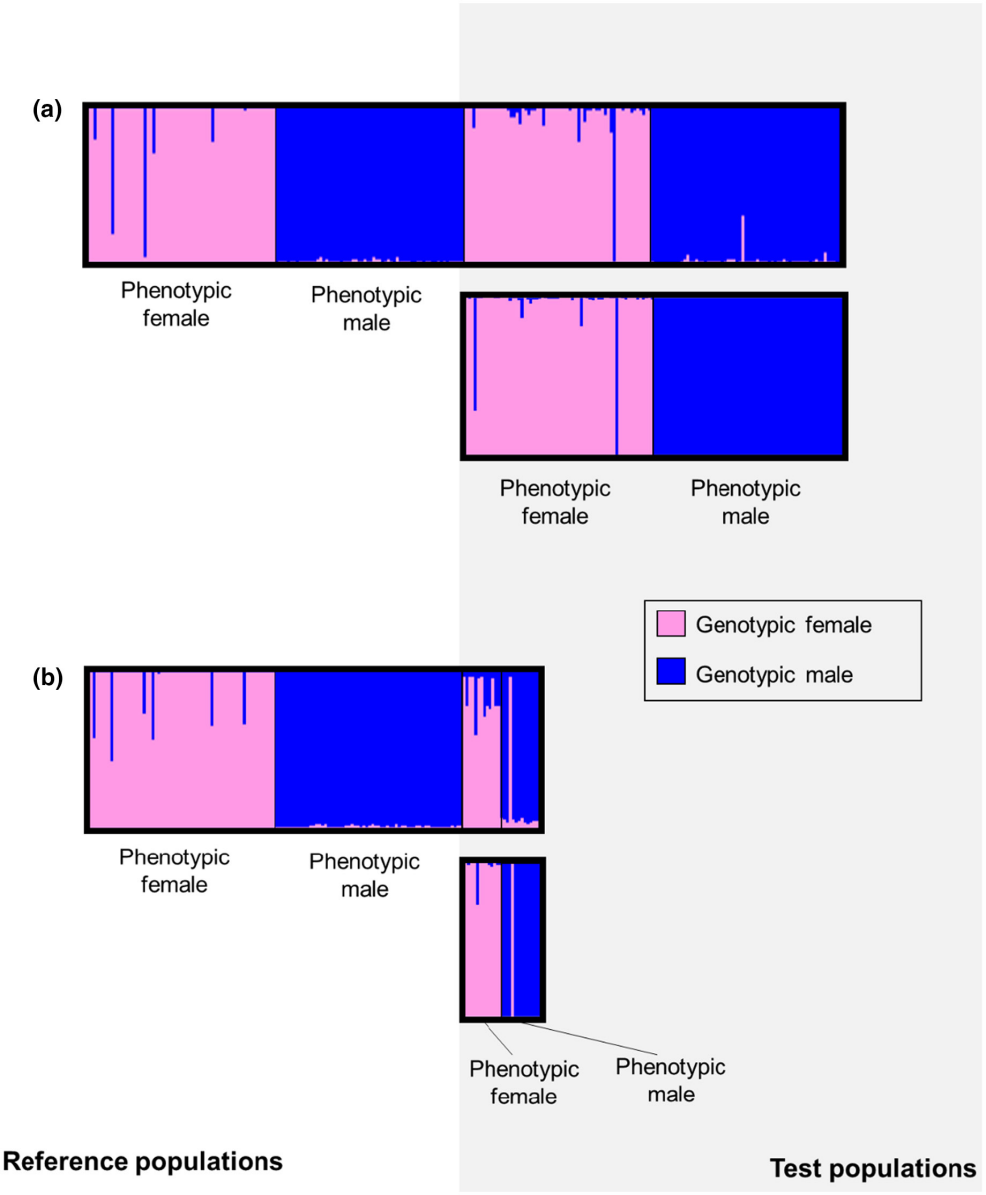
Sex‐associated SNP assignment testing for GT‐seq panel SNP selection, for which the lefthand side (white background) shows reference samples and righthand side (gray background) shows test samples. For both (a) range‐wide validation (*n* = 12 SNPs) and (b) NRM validation only (*n* = 9 SNPs), the top figure shows the Bayesian clustering approach employed by STRUCTURE, and the bottom figure shows the individual assignment approach by Rannala and Mountain (1997) employed by GENECLASS2 (Piry et al., 2004). For all figures, each vertical bar represents the proportion of genetic assignment for an individual to either a genotypic female (pink) or male (blue) cluster, and samples within reference and test groups are grouped by phenotypic sex.

Fourteen putatively sex‐associated SNPs were discovered using RDA and yielded high sex assignment accuracy (Figure 3), two of which were removed due to high missing data or monomorphism in the test samples. Bayesian clustering analyses revealed that the remaining 12 SNPs successfully diagnosed sex in 98.4% of individuals across all combined lineages (Figure 3). We removed one SNP on the tail end of a RADtag and two SNPs monomorphic in the NRM lineage from further consideration for the GT‐seq panel; the remaining nine SNPs demonstrated a 96.2% correct sex assignment rate within just NRM samples (Figure 3). We found that one SNP (Ocp_NRM_1698806_8) diagnosed sex based on heterozygosity with 91.7% accuracy among all combined lineages and 100% accuracy within the NRM lineage. This SNP was located on chromosome 12 (NC_050547.1), which fell within the 3′ untranslated region of the zinc finger transcription factor protein ZNF322.

Of the 155 unique genes associated with robust NRM‐specific outliers, 72 genes demonstrated a putative link to environmental adaptation, for which 56 unique SNPs were annotated and included in the panel. Four SNPs were associated with both PSGs (Sjodin & Russello, 2022) and robust outliers, including one NRM‐specific outlier and three range‐wide outliers. For the elevation‐associated outlier SNPs from Schmidt et al. (2021), 590 unique SNPs exhibited a significant correlation between allele frequency and elevation using linear (*n* = 297) or logistic regression (*n* = 558). After quality filtering, 49 elevation‐associated outlier SNPs that were polymorphic in the NRM lineage were retained for inclusion in the GT‐seq panel.

### 3.2 Panel optimization

SNP selection yielded a total of 818 SNPs for primer design, including 700 neutral loci, nine sex‐associated loci, and 109 putatively adaptive loci (56 robust NRM outliers, four PSG‐associated SNPs, and 49 elevation‐associated variants; Table 3). Primers were successfully designed for 529 of these SNPs. The first test library was composed of all 529 SNPs (447 neutral, nine sex‐associated, and 73 putatively adaptive SNPs) across 85 NRM tissue samples (including eight replicates) and yielded 20,791,752 paired end reads. 50.9% of SNPs genotyped across all individuals (hereafter referred to as “genotyping success”; Table 3). Panel optimization yielded a second test library of 513 SNPs (54.5% genotyping success) and third test library of 446 SNPs (64.5% genotyping success; Table 3). We removed five samples and 136 SNPs with >50% missing data, including two sex‐associated SNPs. Of the four sex‐associated SNPs retained, three were monomorphic in retained samples and removed; the highly diagnostic sex‐associated SNP for NRM samples (Ocp_NRM_1698806_8) remained and genotyped in all retained samples with 0% genotyping error and 94.4% genotyping concordance to RADseq data.

**TABLE 3:** The number of SNPs included in GT‐seq panel design for each category (sex‐associated, neutral, and putatively adaptive) throughout the optimization process of three test libraries, resulting in the final GT‐seq panel to be applied to archival tissue and fecal pellet samples.

Type	Original design	Test library 1	Test library 2	Test library 3	Final
Sex‐associated	9	9	9	6	1
Neutral	700	447	438	386	264
Putatively adaptive					
Elevation‐associated	49	33	29	22	19
PSG‐associated	4	3	3	2	1
Climate‐associated (NRM)	56	37	34	30	22
Total	818	529	513	446	307

The final panel therefore consisted of 307 SNPs (264 neutral, 1 sex‐associated, and 42 putatively adaptive SNPs) with 94.7% genotyping success (mean read depth 324.8x) among 80 retained tissue samples (Table 3). A comparison of genotypes between the retained replicates (*n* = 8) yielded a mean genotyping error of 0.001%. Concordance between RADseq genotypes and GT‐seq genotypes across all retained unique samples and loci was 95.3%.

### 3.3 Panel application to fecal pellet DNA

#### 3.3.1 Genotyping success

**FIGURE 4:**
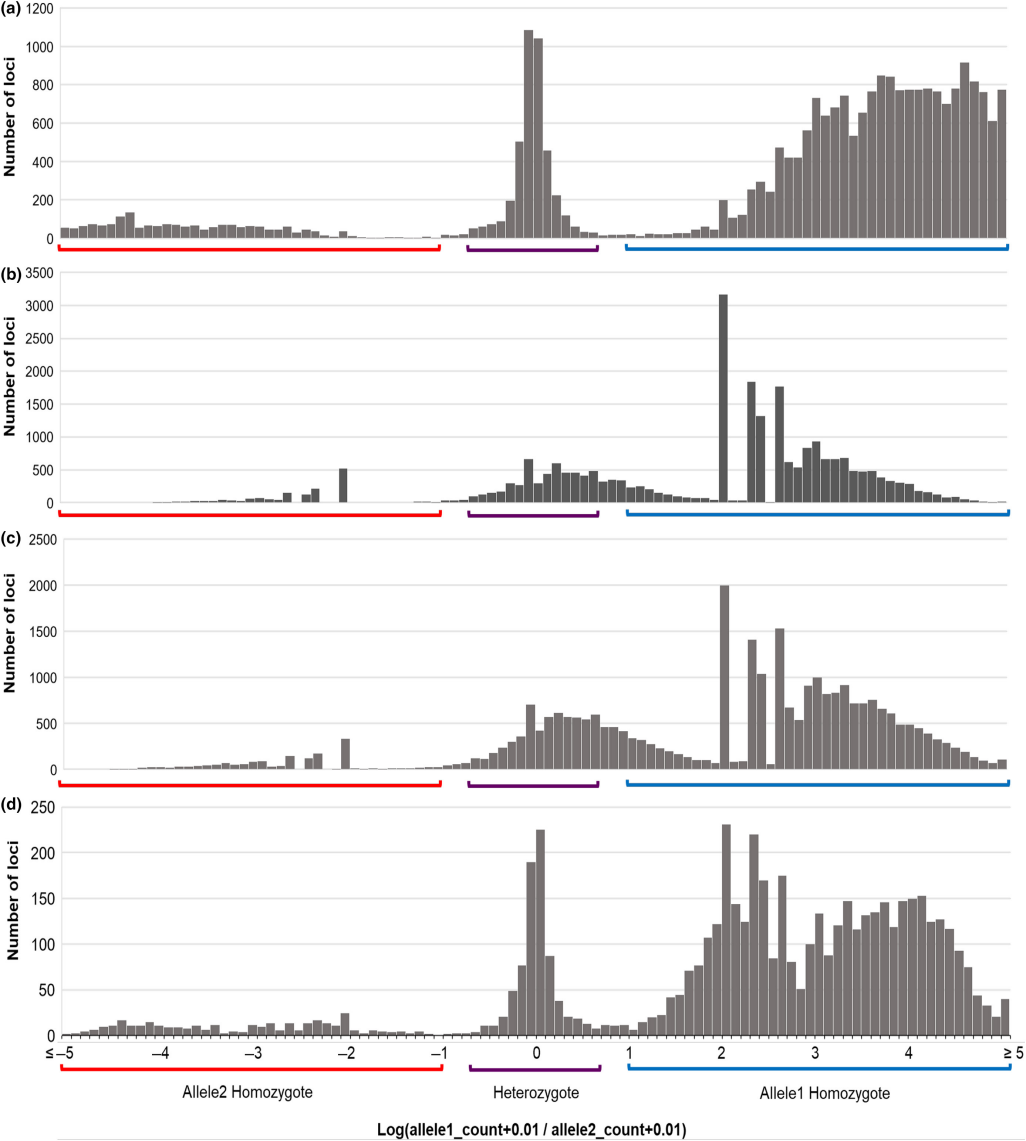
Distribution of the log‐transformed ratio of allele1/allele2 counts for all 307 SNPs across all samples for (a) liver tissue samples from test library 3 (*n* = 85), (b) the first sequencing run of fecal pellet samples (*n* = 143), (c) the second, combined run of fecal pellet samples including the DNA fragment size selection step (*n* = 143), and (d) archival tissue samples (*n* = 18). The minimum/maximum ratio for which allele1 and allele2 homozygotes are called are indicated on the *x* axis in blue (ratio >10:1) and red (ratio <1:10), respectively. The default range of ratios for which a heterozygote is called is indicated in dark purple (1:5–5:1). The width of each vertical bar is 0.1.

The first GT‐seq library of fecal pellet DNA yielded low mean genotyping success (27.2%) at a read depth of 27.5×. Preliminary exploration found that the fecal pellet samples exhibited a broad distribution of allele ratios compared to high‐quality tissue samples among all loci (Figure 4), which prompted us to construct a second GT‐seq library incorporating the DNA size‐selection step (see Section 2.4.1). Combining the reads of both sequencing runs improved genotyping success from 27.2% to 44.7% and raised mean read depth from 27.5× to 84.7× among all loci. Removing SNPs then samples with >50% missing data retained 121 loci (including 105 neutral, one sex‐associated, and 27 putatively adaptive SNPs) across 115 samples with a mean genotyping success of 79.7% (mean read depth 233.1×). Genotyping success was similar between sample years (2021: 78.9%; 2022: 80.4%). Of the 121 loci, 87 were polymorphic with a minor allele frequency >0.03 (including 77 neutral, one sex‐associated, and nine putatively adaptive SNPs; Table 3). Across the eight retained replicate samples and the 87 polymorphic loci, mean genotyping error was found to be 28.4%. Although the putatively adaptive SNPs may prove useful in future spatiotemporal studies, here we focus all further analysis on neutral and sex‐associated SNPs to evaluate the performance of the panel for individual/sex identification, relatedness, and population connectivity.

#### 3.3.2 Efficacy as a monitoring tool

*p*_ID_ and *p*_IDSIBS_ estimates based on the retained neutral SNPs were very low (*p*_ID_ = 1.0E−21; *p*_IDSIBS_ = 1.7E−11), requiring 11 loci for *p*_ID_ < .001 and 21 loci for *p*_IDSIBS_ < .001 (Table B5). Nineteen pairs of “replicated” samples were identified through COLONY. Of these 19 pairs, four of the eight known replicated samples were correctly identified, and one other pair was from the same collection site. The remaining 14 identified “replicate” pairs were composed of samples from different sites. The multi‐locus matches analysis did not identify any replicate samples, despite the inclusion of eight known replicates. The proportion of genotypes shared between known replicates ranged from 52.0% to 86.8% (mean = 71.6%). The estimated rate of relatedness category misclassification among simulated dyads was relatively high for FS (15.6%), HS (34.1%), and unrelated (UR; 18.5%) pairs (Table B6).

No apparent genetic structure was detected among the thirteen sampling sites using either the maximum likelihood clustering approach implemented in ADMIXTURE (Figure B3) or a PCA (Figure B4). The ADMIXTURE clustering approach indicated that all samples most likely belonged to a single population (*K* = 1), though the estimate of *K* = 2 yielded a marginally higher CV error (Figure B3). No pattern was recognized among samples assigned to a particular genetic cluster. Estimates of global (*F*_ST_ = 0.009) and pairwise *F*_ST_ were low, though some significant differences were observed, particularly for site PBO (Table B7). No significant relationship between genetic and geographic distance was detected by the Mantel test (*r* = .046; *p* = .189).

### 3.4 Panel application to archival DNA

Genotyping success of the archival tissue samples was high (84.9%). After filtering for missing data, all samples (*n* = 17 + 1 replicate) and 284 loci were retained; this improved mean genotyping success to 90.5% with a mean read depth of 252.4×, and there was no evidence of genotyping error in the included technical replicate. The sex‐associated SNP correctly assigned the recorded phenotypic sex of the archival specimens with 100% accuracy for samples that genotyped at that locus (15 of 17 individuals). The genotype of one specimen (HS1; accession number CMNMA18736) with no previously recorded sex was genotypically assigned as a female.

## 4 DISCUSSION

Multiplexed targeted amplicon sequencing techniques, such as GT‐seq, can provide cost‐effective approaches for collecting genetic data from low quality and quantity DNA samples, making them potentially useful for long‐term wildlife monitoring using non‐invasive sampling (Burgess et al., 2022; Hayward et al., 2022; Schmidt, Campbell, et al., 2020) and temporal genetic analyses using archival specimens (Setzke et al., 2021). Here, we developed a multi‐purpose GT‐seq panel of 307 SNPs for the NRM lineage of American pikas that was highly successful for genotyping contemporary tissue samples as well as archival skin samples collected up to 93 years ago. Yet, the panel yielded high genotyping error with fecal pellet samples, highlighting potential challenges of applying GT‐seq to fecal DNA from small mammals and suggesting the need for further optimization of sampling strategies, genetic data collection approaches, and/or analysis methods.

During the selection of SNPs for inclusion in the GT‐seq panel, we found that neutral SNP datasets of 350, 700, and over 15,000 SNPs yielded very similar population genetic parameters. Through GT‐seq panel optimization, we aimed to find a balance between maximizing SNP quantity and informativeness while minimizing primer interactions and the additional costs required for a greater number of SNPs. The optimized GT‐seq panel therefore consisted of 307 SNPs (including 264 neutral SNPs, one sex‐associated SNP, and 42 putatively adaptive SNPs). The high genotyping success (94.7%) and low genotyping error (<0.01%) exhibited by liver tissue samples demonstrated the informativeness of the developed GT‐seq panel with contemporary tissue samples and is consistent with the success of recent applications to high‐quality DNA of other species (Burgess et al., 2022; Chang et al., 2021, 2022; Hayward et al., 2022; Sjodin et al., 2020). The high concordance with paired RADseq data (95.3%) further showed the reliability of the developed GT‐seq panel; the few instances of discordance can likely be attributed to differences in genotype calling models or read depth rather than sequencing error (Schmidt, Campbell, et al., 2020).

In contrast, the application of our GT‐seq panel to fecal pellet samples demonstrated the need for further sampling and data collection protocol optimization before it can be reliably applied as a monitoring tool using American pika fecal DNA. Previously, GT‐seq has been successfully applied to minimally and non‐invasively collected samples such as snake cloacal epithelial swabs (Schmidt, Campbell, et al., 2020), deer hair and fecal pellets (Burgess et al., 2022), and polar bear feces (Hayward et al., 2022). Both studies using field‐collected fecal material (Burgess et al., 2022; Hayward et al., 2022) reported high genotyping success (85.6% and 85.1%, respectively) and relatively low genotyping error between replicates (0.0% and 10.7%, respectively). Our genotyping error (28.4%) was substantially greater, while the post‐filtering genotyping success of our fecal pellet samples (79.7%) was relatively comparable. A high proportion of the 307 SNPs genotyped in our final library were filtered out due to missing data or low polymorphism prior to downstream applications, limiting our analyses to a maximum of 77 neutral SNPs. Some fallout due to low polymorphism was anticipated due to the broad geographic range of samples used in SNP discovery compared to the relatively restricted area of application. Yet, the influence of sample DNA quality and quantity may have exacerbated this effect. The frequent misidentification of unique individuals and high misclassification rates among relatedness groups indicated that further work is needed before the developed GT‐seq panel can be used to reliably distinguish individuals and estimate relatedness using American pika fecal pellet DNA. Though the inferred absence of population structure among sites even >65 km apart may be a true signal, previous work has demonstrated low dispersal rates (Peacock, 1997; Peacock & Smith, 1997) and high genetic differentiation among patches located <5 km from each other (Henry et al., 2012; Robson et al., 2016). Our results may therefore be an artifact of the high genotyping error observed, which is known to bias estimates of population structure towards admixture (Kirk & Cardon, 2002; Zhang & Hare, 2012).

This study highlights the need for careful consideration of potential challenges that may be encountered when working with non‐invasive sample materials for applications in conservation genomics. Though our approach of sampling clumps of fresh‐looking pellets from distinct latrines proved adequate for prior microsatellite‐based studies using American pika pellet DNA (Castillo et al., 2014, 2016; Klingler et al., 2023), it is possible that intraspecific contamination may have been a factor in the genotyping error observed. Furthermore, pikas are coprophagous and therefore may ingest their own feces or that of others, resulting in fecal pellet samples potentially containing the DNA of multiple individuals (Castillo & Epps, 2011; Hirakawa, 2001). Despite these considerations, the low individual fuzziness scores (an estimate of among‐sample contamination) calculated using the *Gtseq_Genotyper_v3.pl* genotyping script (https://github.com/GTseq) indicated that inter‐sample contamination was minimal, suggesting that the high genotyping error more likely resulted from issues related to insufficient target species DNA quality/quantity.

Though preliminary quality checks demonstrated the presence of whole genomic DNA extracted from fecal pellets, it is likely that the concentration of target species DNA in these samples was very low. As PCR amplification is a stochastic process, samples with very low starting concentrations of target DNA and/or that contain PCR inhibitors may differentially amplify alleles (Kebschull & Zador, 2015; Kemp et al., 2020), leading to high variance of allele read counts. The broader distribution of allele count ratios within fecal pellet samples compared to tissue samples indicated that differential allele amplification was likely the driving factor of the high genotyping error observed between replicates (Figure 4). The GT‐seq panel development by Hayward et al. (2022) included a qPCR screening step in their library preparation workflow to estimate the concentration of the targeted polar bear DNA in field fecal samples. They found that 65.2% of samples contained no target host DNA, which were subsequently excluded from further analysis. Large discrepancies in genotyping success were also found between replicated samples, suggesting that the concentration of target DNA between subsamples can vary widely (Hayward et al., 2022).

Notably, both prior studies demonstrating high success of GT‐seq with field‐collected fecal samples (Burgess et al., 2022; Hayward et al., 2022) started with a much greater quantity of fecal material than available in this study due to the size difference in target species. Here, we combined up to 12 fecal pellets collected from the same latrine, though the weight of dried fecal samples used for DNA extraction was still approximately one‐tenth of the recommended weight in the manufacturer's protocol (QIAamp® Fast DNA Stool Mini Kit). Future studies using GT‐seq with American pikas or other small mammals may consider combining greater quantities of pellets to further maximize the amount of starting material and target DNA; however, this may increase the risk of introducing more off‐target DNA and/or DNA from different individuals (Castillo et al., 2014; Castillo & Epps, 2011).

The fecal pellets collected in this study may have also varied in DNA quality/quantity and abundance of PCR inhibitors depending on factors affecting pellet decomposition. Such factors may include age (e.g. older pellets may be more deteriorated), composition (e.g. local diet), site and latrine exposure (e.g. degree of shelter from sun and precipitation), and other environmental conditions (e.g. duration frozen and frequency of freeze–thaw cycles; Nichols, 2011). Though efforts were made to sample the freshest pellets, this was relative to their availability at each latrine. We assumed that fresh pellets would yield the least deteriorated DNA (Castillo et al., 2014; Klingler et al., 2023), but they could also contain more active PCR inhibitors from preserved plant material (Monteiro et al., 1997; reviewed in Schrader et al., 2012). Future research exploring the relationship between fecal pellet condition and the quality/quantity of extracted DNA for genomic applications would be highly valuable to assessing the importance of sample quality in overall genotyping success.

The implementation of different sample collection protocols may also maximize the collection of on‐target DNA prior to extraction. In this study, we combined and preserved whole fecal pellets in 100% ethanol prior to extracting DNA from dried and homogenized pellets; however, techniques that recover intestinal epithelial DNA from the surface of fecal material may be more successful by minimizing the impact of contamination. Prior to DNA extraction from American pika fecal pellet samples, Castillo et al. (2016) and Klingler et al. (2023) washed the epithelial cells off whole pellets by inverting tubes containing submerged pellets in lysis buffer solution, which generated adequate DNA concentrations for genotyping microsatellite markers. Ramón‐Laca et al. (2015) described a similar approach of superficially swabbing the outermost layer of mammalian feces to recover epithelial DNA, which demonstrated particular success with herbivores and could be an alternative method to explore. Likewise, Burgess et al. (2022) used only the most superficial “shell” of each pellet prior to DNA extraction and GT‐seq panel application to maximize on‐target DNA. This latter method had been considered for pika fecal pellets in this study, but the small size and absence of a clearly defined outer layer prevented efficient incorporation into the workflow. As various fecal DNA extraction techniques have demonstrated differential success among animal species (Hart et al., 2015; Piggott & Taylor, 2003), exploring other DNA extraction options, such as a MoBio PowerFecal Kit (VWR) or a manual isopropanol extraction method, may also increase DNA recovery.

Additionally, on‐target DNA amplification may be improved by further optimization of PCR1 cycling conditions. The Qiagen Multiplex PCR kit protocol that yielded vast improvements in fecal pellet genotyping success by Burgess et al. (2022) was also found to improve genotyping in this study when compared to the original GT‐seq PCR1 protocol (Campbell et al., 2015) (data not shown). Although the greater number of cycles in the Qiagen protocol likely resulted in a larger quantity of PCR products, this increase would also be expected to create more off‐target amplicons and may introduce further error into the PCR process (Kebschull & Zador, 2015; Lorenz, 2012). Previous genetic studies using American pika fecal pellet DNA to target hypervariable microsatellites constructed a consensus genotype from multiple independent replicates of a single sample (Castillo et al., 2014; Klingler et al., 2023); however, given the known stochasticity of PCR with low quantities of DNA, it may be challenging to assess their accuracy, particularly for biallelic SNPs. Exploring the addition of targeted capture and enrichment techniques within the GT‐seq workflow, including hybridization capture‐based target enrichment methods (reviewed by Jones & Good, 2016; Kozarewa et al., 2015), may also improve on‐target genotyping and read depth at loci of interest. Though sequence capture methods may provide a standalone alternative method to MTAS (e.g. Rapture; Ali et al., 2016), its dependence on previous sequencing efforts, a higher cost per sample, and reduced efficacy with low‐quality DNA may limit its application for long‐term monitoring efforts (reviewed by Meek & Larson, 2019). Genotype accuracy may be further improved by using different genotyping approaches, such as those that employ a probabilistic model instead of hard thresholds to call genotypes, though prior knowledge of genotype distribution within the studied population may be required for optimal accuracy (reviewed in Coble & Bright, 2019; Hendricks et al., 2018). We tested the genotyping model used by Hayward et al. (2022); however, this approach was found to slightly increase mean genotyping error within our dataset and was therefore not investigated further (data not shown).

Aside from protocol modifications to improve target DNA yield and genotyping success using fecal pellet samples, different types of minimally invasive samples may yield greater success with the developed GT‐seq panel. Previous genetic studies have used low‐quality DNA obtained from American pika hair samples (Henry & Russello, 2011, 2013; Lamb et al., 2014; Russello et al., 2015). Although not tested here, the successful application of GT‐seq to deer hair samples (94.1% genotyping success and 0.33% genotyping error; Burgess et al., 2022) sets a precedent for potential use of the developed panel to pika hair samples as an alternative non‐invasive sample material that may also be feasible for long‐term monitoring.

Although similar challenges from low quantity, deteriorated, and contaminated DNA may be anticipated when working with both non‐invasive and archival samples (reviewed in Andrews et al., 2021; Raxworthy & Smith, 2021), our application of the developed GT‐seq panel to museum specimens yielded far greater success than with the fecal pellet samples. Previous work using GT‐seq with archival fish scales (collected from 1973 to 1981; Setzke et al., 2021) demonstrated high genotyping success across 271 SNPs. We found similar success (90.5% genotyping success and 0% genotyping error) using DNA from even older samples (collected in 1930 and 1945), reinforcing the effectiveness of GT‐seq with archival DNA and encouraging future applications. Temporal studies using archival samples have the potential to provide information on long‐term population trends in relation to recorded environmental change (reviewed by Jensen & Leigh, 2022), including comparative analyses of genetic variation between archival and contemporary samples (Hartmann et al., 2014; Jensen et al., 2018; Klingler et al., 2023; Muhlfeld et al., 2014; O'Toole et al., 2020; van der Valk et al., 2019) or within time‐series analyses where long‐term collections are available (Bi et al., 2019; Therkildsen et al., 2013). Considering the interest in monitoring alpine ecosystems and recognition of the American pika as an early sentinel species (Beever et al., 2003; Wilkening & Ray, 2016), such long‐term studies may provide important indications of biological responses to climate change (Klingler et al., 2023). Recently, Klingler et al. (2023) compared the genetic variation and patterns of gene flow between contemporary and archival American pika populations in the Bodie Hills, California (USA) using eight microsatellites. The study documented evidence of genetic erosion and a decrease in among‐site connectivity indicative of the long‐term decline in population size and temporal increase in fragmentation (Klingler et al., 2023). Furthermore, Bi et al. (2019) reconstructed demographic histories and evaluated evidence of directional selection for an alpine chipmunk species (*Tamias alpinus*) using targeted exon sequencing of samples spanning over a century (1911–2012), finding evidence of temporal population fragmentation as well as positive selection at a gene involved in immune response. Together, these studies demonstrate the value of spatiotemporal genomic studies of alpine species experiencing rapid environmental change and highlight the promise of the American pika GT‐seq panel, which includes both neutral and putatively adaptive SNPs, for contributing to such lines of inquiry in the future.

Overall, our study reinforces GT‐seq as an effective tool in conservation genomics using contemporary and archival tissue samples, providing future opportunities for temporal applications using historical specimens almost a century old. Our results further highlight the need for additional optimization of sample and genetic data collection techniques prior to broader‐scale implementation of a non‐invasive genetic monitoring tool for American pikas; though the targeted amplicon sequencing approach employed by GT‐seq has performed well for samples of low quality and quantity DNA in other systems (Burgess et al., 2022; Hayward et al., 2022; Schmidt, Campbell, et al., 2020; Schmidt, Govindarajulu, et al., 2020), we find that the efficacy may remain contingent on species‐specific sample characteristics.

## AUTHOR CONTRIBUTIONS

**Kate E. Arpin:** Conceptualization (supporting); data curation (lead); formal analysis (lead); writing – original draft (lead); writing – review and editing (equal). **Danielle A. Schmidt:** Conceptualization (supporting); data curation (supporting); formal analysis (supporting); writing – review and editing (supporting). **Bryson M. F. Sjodin:** Conceptualization (supporting); writing – review and editing (supporting). **Anthony L. Einfeldt:** Conceptualization (supporting); funding acquisition (supporting); resources (supporting); supervision (supporting); writing – review and editing (supporting). **Kurt Galbreath:** Resources (supporting); writing – review and editing (supporting). **Michael A. Russello:** Conceptualization (lead); funding acquisition (lead); project administration (lead); resources (lead); supervision (lead); writing – original draft (supporting); writing – review and editing (equal).

## CONFLICT OF INTEREST STATEMENT

None declared.

## Supporting information


Appendix S1

## Data Availability

SNP genotypic data are available in the Dryad Digital Repository (https://datadryad.org/stash/share/yew1QiSDlTcrtkpcj7A8rzExSa8g8enkRVJaFmquEII).
