# Supplementary material for: Evaluating genotyping‐in‐thousands by sequencing as a genetic monitoring tool for a climate sentinel mammal using non‐invasive and archival samples
Source: Ecol Evol. 2024 Feb 7;14(2):e10934. doi: 10.1002/ece3.10934 (PMC10850814; doi:10.1002/ece3.10934)
Supplement: Supplementary file 1 — Appendix S1 [file ECE3-14-e10934-s001.docx]

**Appendix A**

**A1. Supporting Methods: Discovering climate-associated outlier SNPs**

*A1.1. Climate Data*

Climate data for 19 bioclimatic variables were downloaded from the WorldClim 2.1 database (Fick and Hijmans, 2017). Following Waterhouse et al. (2018), we then performed a PCA on this dataset to generate uncorrelated climate predictors for use in subsequent outlier detection analysis. The first three PC axes, which explained 88.8% of the climate variation among sampling sites, were retained as composite climate variables.

*A1.2 Outlier Detection*

Latent Factor Mixed Modeling (LFMM) is a univariate regression approach that incorporates hidden latent factors (*K*) into the model to help account for the confounding effects of neutral population structure on overall genetic variation. First, we estimated the number of ancestral populations in our filtered dataset of 23,967 SNPs using the *snmf()* function as implemented in the R package *LEA* (Frichot and François, 2015a). Given evidence for an optimal number of six populations, we then imputed missing data with the *impute()* function using the “mode” method. Next, we ran the *lfmm_ridge* function in the R package *lfmm*, with six latent factors (*K* = 6) to account for underlying population structure. We ran *lfmm* for each PC composite climate variable and subsequently calibrated *p*-values by manually adjusting the genomic inflation factor (λ) following author/manual recommendations (Frichot and François, 2015b). The *p*-values were then adjusted for multiple testing with a Benjamini-Hochberg correction at a false discovery rate of 0.05 to identify outlier SNPs.

Next, we conducted outlier detection using redundancy analysis (RDA; (Forester et al., 2018) as implemented in the R package *vegan* (Oksanen et al., 2016). RDA is a multi-variate ordination approach that examines the association between linear combinations of environmental predictors and SNP genotypes. To facilitate outlier detection, we reduced the 19 climate variables from the WorldClim database down to a set of uncorrelated predictors using the *findCorrelation* function implemented in the *caret* R package (Kuhn et al., 2020). This function calculates pairwise correlations among variables and removes those with absolute correlations greater than 0.7. Using the imputed dataset generated above for LFMM, we identified outliers by running a partial RDA (pRDA) using the six uncorrelated climate variables (bio10, bio15, bio16, bio4, bio7, bio8) and five PCs to control for neutral population structure. Each RDA axis was tested for significance using the *anova.cca* function and those SNPs with loadings three standard deviations from the mean along each significant RDA axis were considered outliers (*p* <0.0027). Those SNPs identified as outliers using both genotype-environment association methods were then considered putatively adaptive variants for inclusion in the SNP panel.

**A2. Supporting Results: Outlier identification**

LFMM analysis identified a total of 1365 outlier SNPs, 572 of which were associated with PC1, 408 were associated with PC2, and 385 were associated with PC3. For the pRDA, the first 3 axes were significant (*p* = 0.001); there were 254 outliers associated with RDA1, 334 associated with RDA2, and 547 associated with RDA3. Across methods, there were 155 robust outliers that were evaluated for inclusion in the panel.

**Appendix B**

Supplementary tables and figures, including supporting results for comparing neutral SNP datasets in GT-seq panel design, validating sex-associated SNP selection, and evaluating the panel as a monitoring tool with fecal pellet samples.

**Table B1.** Metadata for archival skin tissue samples and liver tissue samples (collected by Galbreath et al., 2009), including museum (Cowan Vertebrate Museum, Canadian Museum of Nature, or Cornell University Museum of Vertebrates) and accession numbers, specimen sex, sample collector, and date and coordinates of sample collection.

| **Sample ID** | **Museum** | **Accession #** | **Sex** | **Sample type** | **Collector** | **Collection date** | **Latitude Longitude**  **(Decimal degrees)** |
| --- | --- | --- | --- | --- | --- | --- | --- |
| HA1 | Cowan | M001587 | female | Skin | James Hatter | 1945-09-03 | 52.7180555 N 118.2677778 W |
| HA2 | Cowan | M001591 | male | Skin | James Hatter | 1945-09-06 | 52.7180555 N 118.2677778 W |
| HA3 | Cowan | M001592 | female | Skin | James Hatter | 1945-09-06 | 52.7180555 N 118.2677778 W |
| HS1 | CMN | CMNMA 18736 | missing | Skin | Austin L. Rand | 1945-08-31 | 52.216667 N 117.166667 W |
| HS2 | CMN | CMNMA 18743 | male | Skin | H.P. Clemens | 1945-09-01 | 52.216667 N 117.166667 W |
| HS3 | CMN | CMNMA 18744 | female | Skin | H.P. Clemens | 1945-09-01 | 52.216667 N 117.166667 W |
| HV1 | CMN | CMNMA 10780 | female | Skin | Ian McTaggart | 1930-07-10 | 52.733333 N 118.333333 W |
| HV2 | CMN | CMNMA 10781 | male | Skin | Ian McTaggart | 1930-07-10 | 52.733333 N 118.333333 W |
| HV3 | CMN | CMNMA 10782 | female | Skin | Ian McTaggart | 1930-07-10 | 52.733333 N 118.333333 W |
| HV4 | CMN | CMNMA 10783 | male | Skin | Ian McTaggart | 1930-07-10 | 52.733333 N 118.333333 W |
| HV5 | CMN | CMNMA 10784 | female | Skin | Ian McTaggart | 1930-07-10 | 52.733333 N 118.333333 W |
| HV6 | CMN | CMNMA 10798 | male | Skin | Ian McTaggart | 1930-07-14 | 52.733333 N 118.333333 W |
| HV7 | CMN | CMNMA 10813 | male | Skin | Ian McTaggart | 1930-07-18 | 52.733333 N 118.333333 W |
| HV8 | CMN | CMNMA 10814 | male | Skin | Ian McTaggart | 1930-07-18 | 52.733333 N 118.333333 W |
| HV9 | CMN | CMNMA 10815 | male | Skin | Ian McTaggart | 1930-07-18 | 52.733333 N 118.333333 W |
| HV10 | CMN | CMNMA 10816 | male | Skin | Ian McTaggart | 1930-07-18 | 52.733333 N 118.333333 W |
| HV11 | CMN | CMNMA 10817 | female | Skin | Ian McTaggart | 1930-07-18 | 52.733333 N 118.333333 W |
| KG263a | CUMV | CUMV 20237 | male | Liver | Kurt Galbreath et al. | 2004-07-27 | 46.0299 N 114.2923 W |
| KG264a | CUMV | CUMV 20238 | female | Liver | Kurt Galbreath et al. | 2004-07-27 | 46.0299 N 114.2923 W |
|  |  |  |  |  |  |  |  |
|  |  |  |  |  |  |  |  |
| **Table B1.** Continued. | | | | | | | |
| **Sample ID** | **Museum** | **Accession #** | **Sex** | **Sample type** | **Collector** | **Collection date** | **Latitude Longitude**  **(Decimal degrees)** |
| KG265 | CUMV | CUMV 20239 | male | Liver | Kurt Galbreath et al. | 2004-07-27 | 46.0299 N 114.2923 W |
| KG266a | CUMV | CUMV 20240 | male | Liver | Kurt Galbreath et al. | 2004-07-27 | 46.0299 N 114.2923 W |
| KG268 | CUMV | CUMV 20241 | female | Liver | Kurt Galbreath et al. | 2004-07-29 | 44.3115997314 N 113.8961029053 W |
| KG269 | CUMV | CUMV 20242 | male | Liver | Kurt Galbreath et al. | 2004-07-29 | 44.3115997314 N 113.8961029053 W |
| KG270 | CUMV | CUMV 20243 | female | Liver | Kurt Galbreath et al. | 2004-07-30 | 44.3115997314 N 113.8961029053 W |
| KG271 | CUMV | CUMV 20244 | male | Liver | Kurt Galbreath et al. | 2004-07-30 | 44.3115997314 N 113.8961029053 W |
| KG272 | CUMV | CUMV 20245 | female | Liver | Kurt Galbreath et al. | 2004-07-30 | 44.3115997314 N 113.8961029053 W |
| KG273 | CUMV | CUMV 20246 | male | Liver | Kurt Galbreath et al. | 2004-07-30 | 44.3115997314 N 113.8961029053 W |
| KG274 | CUMV | CUMV 20247 | male | Liver | Kurt Galbreath et al. | 2004-07-30 | 44.3115997314 N 113.8961029053 W |
| KG275 | CUMV | CUMV 20248 | female | Liver | Kurt Galbreath et al. | 2004-07-31 | 43.6155014038 N 115.4350967407 W |
| KG276 | CUMV | CUMV 20249 | female | Liver | Kurt Galbreath et al. | 2004-07-31 | 43.6155014038 N 115.4350967407 W |
| KG277 | CUMV | CUMV 20250 | female | Liver | Kurt Galbreath et al. | 2004-07-31 | 43.6155014038 N 115.4350967407 W |
| KG278 | CUMV | CUMV 20251 | female | Liver | Kurt Galbreath et al. | 2004-07-31 | 43.6155014038 N 115.4350967407 W |
| KG280 | CUMV | CUMV 20252 | male | Liver | Kurt Galbreath et al. | 2004-07-31 | 43.6155014038 N 115.4350967407 W |
| KG281 | CUMV | CUMV 20253 | male | Liver | Kurt Galbreath et al. | 2004-08-01 | 43.6155014038 N 115.4350967407 W |
| KG282 | CUMV | CUMV 20254 | male | Liver | Kurt Galbreath et al. | 2004-08-01 | 43.6155014038 N 115.4350967407 W |
| KG283a | CUMV | CUMV 20255 | male | Liver | Kurt Galbreath et al. | 2004-08-02 | 45.1870002747 N 116.5752029419 W |
| KG284 | CUMV | CUMV 20256 | female | Liver | Kurt Galbreath et al. | 2004-08-02 | 45.1870002747 N 116.5752029419 W |
| KG285 | CUMV | CUMV 20257 | male | Liver | Kurt Galbreath et al. | 2004-08-02 | 45.1870002747 N 116.5752029419 W |
| KG286 | CUMV | CUMV 20258 | female | Liver | Kurt Galbreath et al. | 2004-08-02 | 45.1870002747 N 116.5752029419 W |
| KG287 | CUMV | CUMV 20259 | male | Liver | Kurt Galbreath et al. | 2004-08-02 | 45.1870002747 N 116.5752029419 W |
| KG288 | CUMV | CUMV 20260 | female | Liver | Kurt Galbreath et al. | 2004-08-02 | 45.1870002747 N 116.5752029419 W |
| KG289 | CUMV | CUMV 20261 | male | Liver | Kurt Galbreath et al. | 2004-08-03 | 45.1827011108 N 116.5641021729 W |
| KG290 | CUMV | CUMV 20262 | female | Liver | Kurt Galbreath et al. | 2004-08-03 | 45.1827011108 N 116.5641021729 W |
| KG291 | CUMV | CUMV 20263 | male | Liver | Kurt Galbreath et al. | 2004-08-03 | 45.1827011108 N 116.5641021729 W |
| KG294 | CUMV | CUMV 20266 | male | Liver | Kurt Galbreath et al. | 2004-08-03 | 45.1827011108 N 116.5641021729 W |
| KG295 | CUMV | CUMV 20267 | female | Liver | Kurt Galbreath et al. | 2004-08-03 | 45.1827011108 N 116.5641021729 W |
|  |  |  |  |  |  |  |  |
|  |  |  |  |  |  |  |  |
| **Table B1.** Continued. | | | | | | | |
| **Sample ID** | **Museum** | **Accession #** | **Sex** | **Sample type** | **Collector** | **Collection date** | **Latitude Longitude**  **(Decimal degrees)** |
| KG296 | CUMV | CUMV 20268 | male | Liver | Kurt Galbreath et al. | 2004-08-03 | 45.1827011108 N 116.5641021729 W |
| KG298 | CUMV | CUMV 20270 | female | Liver | Kurt Galbreath et al. | 2004-08-06 | 48.6316986084 N 116.5705032349 W |
| KG299 | CUMV | CUMV 20271 | female | Liver | Kurt Galbreath et al. | 2004-08-07 | 48.6315002441 N 116.5717010498 W |
| KG300 | CUMV | CUMV 20272 | male | Liver | Kurt Galbreath et al. | 2004-08-07 | 48.6315002441 N 116.5717010498 W |
| KG301 | CUMV | CUMV 20273 | female | Liver | Kurt Galbreath et al. | 2004-08-07 | 48.6315002441 N 116.5717010498 W |
| KG302 | CUMV | CUMV 20274 | male | Liver | Kurt Galbreath et al. | 2004-08-07 | 48.6315002441 N 116.5717010498 W |
| KG303 | CUMV | CUMV 20275 | male | Liver | Kurt Galbreath et al. | 2004-08-07 | 48.6315002441 N 116.5717010498 W |
| KG304 | CUMV | CUMV 20276 | male | Liver | Kurt Galbreath et al. | 2004-08-07 | 48.6315002441 N 116.5717010498 W |
| KG305 | CUMV | CUMV 20277 | female | Liver | Kurt Galbreath et al. | 2004-08-07 | 48.6315002441 N 116.5717010498 W |
| KG306 | CUMV | CUMV 20062 | male | Liver | Kurt Galbreath et al. | 2004-08-08 | 48.6307983398 N 116.5886001587 W |
| KG307 | CUMV | CUMV 20278 | male | Liver | Kurt Galbreath et al. | 2004-08-08 | 48.6307983398 N 116.5886001587 W |
| KG308 | CUMV | CUMV 20279 | male | Liver | Kurt Galbreath et al. | 2004-08-08 | 48.6307983398 N 116.5886001587 W |
| KG309 | CUMV | CUMV 20280 | female | Liver | Kurt Galbreath et al. | 2004-08-08 | 48.6307983398 N 116.5886001587 W |
| KG310 | CUMV | CUMV 20281 | male | Liver | Kurt Galbreath et al. | 2004-08-08 | 48.6307983398 N 116.5886001587 W |
| KG311 | CUMV | CUMV 20282 | male | Liver | Kurt Galbreath et al. | 2004-08-08 | 48.6307983398 N 116.5886001587 W |
| KG401 | CUMV | CUMV 20591 | male | Liver | Kurt Galbreath et al. | 2005-07-20 | 52.0372009277 N 116.5156021118 W |
| KG402 | CUMV | CUMV 20696 | female | Liver | Kurt Galbreath et al. | 2005-07-07 | 52.0372009277 N 116.5156021118 W |
| KG404a | CUMV | CUMV 20697 | female | Liver | Kurt Galbreath et al. | 2005-07-08 | 52.0603981018 N 116.5214996338 W |
| KG406 | CUMV | CUMV 20699 | female | Liver | Kurt Galbreath et al. | 2005-07-09 | 52.0579986572 N 116.5413970947 W |
| KG407 | CUMV | CUMV 20700 | male | Liver | Kurt Galbreath et al. | 2005-07-09 | 52.0579986572 N 116.5413970947 W |
| KG408 | CUMV | CUMV 20701 | male | Liver | Kurt Galbreath et al. | 2005-07-09 | 52.0579986572 N 116.5413970947 W |
| KG409 | CUMV | CUMV 20702 | female | Liver | Kurt Galbreath et al. | 2005-07-09 | 52.0579986572 N 116.5413970947 W |
| KG410 | CUMV | CUMV 20703 | male | Liver | Kurt Galbreath et al. | 2005-07-09 | 52.0579986572 N 116.5413970947 W |
| KG411 | CUMV | CUMV 20704 | male | Liver | Kurt Galbreath et al. | 2005-07-09 | 52.0579986572 N 116.5413970947 W |
| KG412 | CUMV | CUMV 20705 | male | Liver | Kurt Galbreath et al. | 2005-07-09 | 52.0579986572 N 116.5413970947 W |
| KG413b | CUMV | CUMV 20706 | male | Liver | Kurt Galbreath et al. | 2005-07-09 | 52.0579986572 N 116.5413970947 W |
| KG415 | CUMV | CUMV 20707 | male | Liver | Kurt Galbreath et al. | 2005-07-10 | 52.0574989319 N 116.4794006348 W |
|  |  |  |  |  |  |  |  |
|  |  |  |  |  |  |  |  |
| **Table B1.** Continued. | | | | | | | |
| **Sample ID** | **Museum** | **Accession #** | **Sex** | **Sample type** | **Collector** | **Collection date** | **Latitude Longitude**  **(Decimal degrees)** |
| KG416 | CUMV | CUMV 20708 | male | Liver | Kurt Galbreath et al. | 2005-07-10 | 52.0574989319 N 116.4794006348 W |
| KG417 | CUMV | CUMV 20709 | male | Liver | Kurt Galbreath et al. | 2005-07-10 | 52.0574989319 N 116.4794006348 W |
| KG418 | CUMV | CUMV 20710 | female | Liver | Kurt Galbreath et al. | 2005-07-10 | 52.0574989319 N 116.4794006348 W |
| KG419 | CUMV | CUMV 20711 | male | Liver | Kurt Galbreath et al. | 2005-07-10 | 52.0574989319 N 116.4794006348 W |
| KG420 | CUMV | CUMV 21159 | female | Liver | Kurt Galbreath et al. | 2005-07-10 | 52.0574989319 N 116.4794006348 W |
| KG421 | CUMV | CUMV 20590 | male | Liver | Kurt Galbreath et al. | 2005-07-14 | 53.3591003418 N 120.1137008667 W |
| KG422 | CUMV | CUMV 20712 | female | Liver | Kurt Galbreath et al. | 2005-07-14 | 53.3652992249 N 120.1249008179 W |
| KG423 | CUMV | CUMV 20713 | male | Liver | Kurt Galbreath et al. | 2005-07-14 | 53.3652992249 N 120.1249008179 W |
| KG424 | CUMV | CUMV 20718 | male | Liver | Kurt Galbreath et al. | 2005-07-14 | 53.3652992249 N 120.1249008179 W |
| KG425 | CUMV | CUMV 20714 | male | Liver | Kurt Galbreath et al. | 2005-07-14 | 53.3586006165 N 120.1234970093 W |
| KG427 | CUMV | CUMV 20716 | male | Liver | Kurt Galbreath et al. | 2005-07-15 | 53.3482017517 N 120.1358032227 W |
| KG428 | CUMV | CUMV 20717 | male | Liver | Kurt Galbreath et al. | 2005-07-15 | 53.3482017517 N 120.1358032227 W |
| KG430 | CUMV | CUMV 20589 | male | Liver | Kurt Galbreath et al. | 2005-07-20 | 54.0773010254 N 119.418800354 W |
| KG432 | CUMV | CUMV 20721 | male | Liver | Kurt Galbreath et al. | 2005-07-20 | 54.0773010254 N 119.418800354 W |
| KG435 | CUMV | CUMV 20724 | male | Liver | Kurt Galbreath et al. | 2005-07-20 | 54.0773010254 N 119.418800354 W |
| KG478 | CUMV | CUMV 20769 | female | Liver | Kurt Galbreath et al. | 2005-08-02 | 51.7242012024 N 119.852897644 W |
| KG479b | CUMV | CUMV 20580 | male | Liver | Kurt Galbreath et al. | 2005-08-20 | 51.7177009583 N 119.8623962402 W |
| KG480 | CUMV | CUMV 20756 | female | Liver | Kurt Galbreath et al. | 2005-08-02 | 51.7242012024 N 119.852897644 W |
| KG481 | CUMV | CUMV 20757 | male | Liver | Kurt Galbreath et al. | 2005-08-02 | 51.7242012024 N 119.852897644 W |
| KG482a | CUMV | CUMV 20770 | male | Liver | Kurt Galbreath et al. | 2005-08-02 | 51.7242012024 N 119.852897644 W |
| KG486a | CUMV | CUMV 20772 | male | Liver | Kurt Galbreath et al. | 2005-08-02 | 51.7242012024 N 119.852897644 W |

**Table B2.** List of all datasets used to select neutral, sex-associated, and putatively adaptive SNPs for GT-seq panel design, including the number of SNPs and samples, genomic lineages included, sampling year, and any collaborations providing samples or data used in this research.

| **Dataset #** | **Description** | |  |  |  |  |
| --- | --- | --- | --- | --- | --- | --- |
| 1 | RADseq data of all SNPs and samples range-wide: Provided data for Datasets 1-9, 12 | | | | | |
|  | *No. SNPs* | *No. samples* | *Genomic lineage(s)* | *Sampling year* | *Citation* | *Provided SNPs for GT-seq panel?* |
|  | Varied based on filtering parameters | 366 | Range-wide | 2004-2005 | Samples from Galbreath et al. (2009) | - |
| 2 | Full neutral SNP dataset: Contained all quality-filtered SNPs for comparing estimates of individual identification, relatedness, and population structure to Datasets 3 and 4 | | | | | |
|  | *No. SNPs* | *No. samples* | *Genomic lineage(s)* | *Sampling year* | *Citation* | *Provided SNPs for GT-seq panel?* |
|  | 15,480 | 77 | NRM | 2004-2005 | Samples from Galbreath et al. (2009) | - |
| 3 | 700 neutral SNP subset: A subset of Dataset 2 for comparing estimates of individual identification, relatedness, and population structure to Datasets 2 and 4 | | | | | |
|  | *No. SNPs* | *No. samples* | *Genomic lineage(s)* | *Sampling year* | *Citation* | *Provided SNPs for GT-seq panel?* |
|  | 700 | 77 | NRM | 2004-2005 | Samples from Galbreath et al. (2009) | 700 |
| 4 | 350 neutral SNP subset: A subset of Dataset 3 for comparing estimates of individual identification, relatedness, and population structure to Datasets 2 and 3 | | | | | |
|  | *No. SNPs* | *No. samples* | *Genomic lineage(s)* | *Sampling year* | *Citation* | *Provided SNPs for GT-seq panel?* |
|  | 350 | 77 | NRM | 2004-2005 | Samples from Galbreath et al. (2009) | - |
|  |  | | | | | |
|  |  | | | | | |
| **Table B2.** | Continued. | | | | | |
| **Dataset #** | **Dataset #** | | | | | |
| 5 | Reference dataset of sex-associated SNPs: Used to discover sex-associated SNPs using a redundancy analysis | | | | | |
|  | *No. SNPs* | *No. samples* | *Genomic lineage(s)* | *Sampling year* | *Citation* | *Provided SNPs for GT-seq panel?* |
|  | 14 | 128 | Range-wide | 2004-2005 | Samples from Galbreath et al. (2009) | - |
| 6 | Test dataset of sex-associated SNPs (range-wide): Used to validate the sexing ability of identified sex-associated SNPs across all combined lineages using Bayesian clustering and individual assignment analyses | | | | | |
|  | *No. SNPs* | *No. samples* | *Genomic lineage(s)* | *Sampling year* | *Citation* | *Provided SNPs for GT-seq panel?* |
|  | 12 | 128 | Range-wide | 2004-2005 | Samples from Galbreath et al. (2009) | - |
| 7 | Test dataset of sex-associated SNPs (NRM only): Used to validate the sexing ability of identified sex-associated SNPs within NRM lineage using Bayesian clustering and individual assignment analyses | | | | | |
|  | *No. SNPs* | *No. samples* | *Genomic lineage(s)* | *Sampling year* | *Citation* | *Provided SNPs for GT-seq panel?* |
|  | 9 | 26 | NRM | 2004-2005 | Samples from Galbreath et al. (2009) | 9 |
| 8 | Putatively adaptive climate-associated robust outliers (range-wide): Solely for comparison to Dataset 10 | | | | | |
|  | *No. SNPs* | *No. samples* | *Genomic lineage(s)* | *Sampling year* | *Citation* | *Provided SNPs for GT-seq panel?* |
|  | 599 | 366 | Range-wide | 2004-2005 | Samples from Galbreath et al. (2009); data from Schmidt et al. (submitted) | - |
|  |  | | | | | |
| **Table B2.** Continued. | | | | | | |
| **Dataset #** | **Description** | | | | | |
| 9 | Putatively adaptive climate-associated robust outliers (NRM only): Outliers that demonstrated evidence of association with climate adaptation in the literature were included in the panel | | | | | |
|  | *No. SNPs* | *No. samples* | *Genomic lineage(s)* | *Sampling year* | *Citation* | *Provided SNPs for GT-seq panel?* |
|  | 155 | 77 | NRM | 2004-2005 | Samples from Galbreath et al. (2009); data from Schmidt et al. (submitted) | 56 |
| 10 | Putatively adaptive SNPs associated with positively selected genes (PSGs): PSG-associated SNPs that were also present in Datasets 8 or 9 were included in the panel | | | | | |
|  | *No. genes* | *No. samples* | *Genomic lineage(s)* | *Sampling year* | *Citation* | *Provided SNPs for GT-seq panel?* |
|  | 41 | n/a | n/a | n/a | Data from Sjodin and Russello (2022) | 4 |
| 11 | Putatively adaptive elevation-associated SNPs: Original dataset of elevation-associated SNPs used to conduct linear and logistic regressions with elevation to identify significant relationships | | | | | |
|  | *No. genes* | *No. samples* | *Genomic lineage(s)* | *Sampling year* | *Citation* | *Provided SNPs for GT-seq panel?* |
|  | 899 | 92 | Cascades | 2015 | Data from Schmidt et al. (2021) | - |
| 12 | Putatively adaptive elevation-associated SNPs in NRM lineage: SNPs from Dataset 11 significantly associated with elevation were genotyped and quality-filtered with samples from NRM | | | | | |
|  | *No. genes* | *No. samples* | *Genomic lineage(s)* | *Sampling year* | *Citation* | *Provided SNPs for GT-seq panel?* |
|  | 590 | 77 | NRM | 2004-2005 | Samples from Galbreath et al. (2009) | 49 |

**Table B3****.** Relatedness misclassification rates between neutral SNP datasets for GT-seq panel SNP selection calculated using the R package *irelr* (Gonçalves da Silva and Russello, 2011) by simulating 1,000,000 dyads for each relatedness category (FS = full sibling; HS = half-sibling; UR = unrelated).

|  |  |  | **Proportion of row category classed as each** | | |
| --- | --- | --- | --- | --- | --- |
|  |  |  | **FS** | **HS** | **UR** |
| **350 SNPs** | **Simulated category** | **FS** | 0.9715 | 0.0285 | 0.0000 |
|  |  | **HS** | 0.0285 | 0.9250 | 0.0465 |
|  |  | **UR** | 0.0000 | 0.0465 | 0.9535 |
| **700 SNPs** | **Simulated category** | **FS** | 0.9961 | 0.0039 | 0.0000 |
|  |  | **HS** | 0.0040 | 0.9865 | 0.0096 |
|  |  | **UR** | 0.0000 | 0.0095 | 0.9905 |
| **15480 SNPs** | **Simulated category** | **FS** | 1.0000 | 0.0000 | 0.0000 |
|  |  | **HS** | 0.0000 | 1.0000 | 0.0000 |
|  |  | **UR** | 0.0000 | 0.0000 | 1.0000 |

**Table B****4.** Among-site pairwise *F*_ST_ of neutral SNP datasets for GT-seq panel SNP selection, computed using the R package *hierfstat* (Goudet, 2005). Values that deviate significantly from zero (*p* = 0.05) using 1000 bootstraps are bolded. See Fig. 2 for geographic locations of sampling sites.

|  |  |  |  |  | **Sites** |  |  |  |  |  |
| --- | --- | --- | --- | --- | --- | --- | --- | --- | --- | --- |
|  |  | 3 | 4 | 5 | 6 | 13 | 14 | 15 | 16 | 17 |
| **350 SNPs** | 3 |  | - | - | - | - | - | - | - | - |
|  | 4 | **0.3330** |  | - | - | - | - | - | - | - |
|  | 5 | **0.1634** | **0.3664** |  | - | - | - | - | - | - |
|  | 6 | **0.1882** | **0.2270** | **0.2174** |  | - | - | - | - | - |
|  | 13 | **0.4258** | **0.3488** | **0.4688** | **0.2922** |  | - | - | - | - |
|  | 14 | **0.4013** | **0.3190** | **0.4180** | **0.2591** | **0.2548** |  | - | - | - |
|  | 15 | **0.4483** | **0.3738** | **0.4812** | **0.3128** | **0.3366** | **0.2401** |  | - | - |
|  | 16 | **0.4044** | **0.3209** | **0.4267** | **0.2590** | **0.2599** | **0.1894** | **0.2091** |  | - |
|  | 17 | **0.3592** | **0.2848** | **0.4006** | **0.2075** | **0.2152** | **0.1318** | **0.2184** | **0.1215** |  |
| **700 SNPs** | 3 |  | - | - | - | - | - | - | - | - |
|  | 4 | **0.3409** |  | - | - | - | - | - | - | - |
|  | 5 | **0.1797** | **0.4087** |  | - | - | - | - | - | - |
|  | 6 | **0.1725** | **0.2628** | **0.2305** |  | - | - | - | - | - |
|  | 13 | **0.4048** | **0.3419** | **0.4583** | **0.2878** |  | - | - | - | - |
|  | 14 | **0.3850** | **0.3370** | **0.4235** | **0.2764** | **0.2641** |  | - | - | - |
|  | 15 | **0.4390** | **0.3747** | **0.4807** | **0.3274** | **0.3173** | **0.2549** |  | - | - |
|  | 16 | **0.3850** | **0.3325** | **0.4346** | **0.2732** | **0.2589** | **0.2009** | **0.1966** |  | - |
|  | 17 | **0.3319** | **0.2773** | **0.3929** | **0.2190** | **0.2019** | **0.1393** | **0.1969** | **0.1214** |  |
| **15480 SNPs** | 3 |  | - | - | - | - | - | - | - | - |
|  | 4 | **0.3405** |  | - | - | - | - | - | - | - |
|  | 5 | **0.1777** | **0.3988** |  | - | - | - | - | - | - |
|  | 6 | **0.1764** | **0.2594** | **0.2186** |  | - | - | - | - | - |
|  | 13 | **0.3989** | **0.3378** | **0.4377** | **0.2931** |  | - | - | - | - |
|  | 14 | **0.3813** | **0.3505** | **0.4184** | **0.2762** | **0.2739** |  | - | - | - |
|  | 15 | **0.4200** | **0.3935** | **0.4600** | **0.3135** | **0.3142** | **0.2369** |  | - | - |
|  | 16 | **0.3981** | **0.3710** | **0.4393** | **0.2906** | **0.2903** | **0.2124** | **0.1863** |  | - |
|  | 17 | **0.3237** | **0.2871** | **0.3649** | **0.2043** | **0.2051** | **0.1502** | **0.1872** | **0.1578** |  |

| **Site** | ***n*** | ***P*_ID_** | ***P*_IDSIBS_** |
| --- | --- | --- | --- |
| **PWO** | 8 | 5.0E-21 | 3.6E-11 |
| **PWP** | 5 | 1.3E-21 | 1.9E-11 |
| **PMA** | 10 | 9.9E-20 | 1.9E-10 |
| **PST** | 7 | 2.1E-21 | 2.8E-11 |
| **PLO** | 8 | 3.3E-20 | 1.1E-10 |
| **PSP** | 11 | 6.8E-19 | 4.8E-10 |
| **PCL** | 12 | 9.4E-20 | 1.5E-10 |
| **PPL** | 5 | 6.7E-21 | 5.0E-11 |
| **PBO** | 9 | 3.5E-18 | 9.0E-10 |
| **PH** | 6 | 7.9E-20 | 1.6E-10 |
| **PGS** | 8 | 6.1E-21 | 4.3E-11 |
| **PIL** | 10 | 2.2E-20 | 8.8E-11 |
| **PLA** | 8 | 1.7E-20 | 6.2E-11 |
| **Combined** | 107 | 1.0E-21 | 1.7E-11 |

**Table B5.** *P*_ID_ and *P*_IDSIBS_ estimates using GenAlEx v. 6.5 of fecal pellets among nine sites.

**Table B6.** Relatedness misclassification rates estimated using the R package *irelr* (Gonçalves da Silva and Russello, 2011) with 1,000,000 simulated dyads for each relatedness category (FS = full sibling, HS = half sibling, UR = unrelated) for fecal pellet samples.

|  |  | **Proportion of row category classed as each** | | |
| --- | --- | --- | --- | --- |
|  |  | **FS** | **HS** | **UR** |
| **Simulated category** | **FS** | 0.8438 | 0.1538 | 0.0023 |
|  | **HS** | 0.1562 | 0.6591 | 0.1847 |
|  | **UR** | 0.0033 | 0.1814 | 0.8153 |

**Table B7.** Among-site pairwise and global *F*_ST_ using the R package *hierfstat* (Goudet, 2005) for fecal pellet samples. Values that deviated significantly from zero (*p* = 0.05) using 1000 bootstraps are bolded.

|  | **PBO** | **PCL** | **PGS** | **PH** | **PIL** | **PLA** | **PLO** | **PMA** | **PPL** | **PSP** | **PST** | **PWO** | **PWP** | **Global *F*_ST_** |
| --- | --- | --- | --- | --- | --- | --- | --- | --- | --- | --- | --- | --- | --- | --- |
| **PBO** |  | - | - | - | - | - | - | - | - | - | - | - | - | 0.009 |
| **PCL** | 0.011 |  | - | - | - | - | - | - | - | - | - | - | - |  |
| **PGS** | 0.010 | -0.003 |  | - | - | - | - | - | - | - | - | - | - |  |
| **PH** | **0.032** | -0.007 | **-0.031** |  | - | - | - | - | - | - | - | - | - |  |
| **PIL** | 0.012 | 0.005 | -0.020 | -0.004 |  | - | - | - | - | - | - | - | - |  |
| **PLA** | **0.034** | 0.008 | 0.007 | **0.030** | 0.018 |  | - | - | - | - | - | - | - |  |
| **PLO** | **0.025** | 0.005 | 0.009 | -0.006 | **0.023** | 0.013 |  | - | - | - | - | - | - |  |
| **PMA** | **0.028** | -0.009 | -0.008 | -0.014 | 0.003 | 0.005 | -0.001 |  | - | - | - | - | - |  |
| **PPL** | 0.011 | 0.017 | -0.010 | **0.030** | 0.015 | 0.003 | 0.015 | 0.006 |  | - | - | - | - |  |
| **PSP** | 0.006 | -0.003 | -0.005 | 0.006 | 0.003 | -0.003 | 0.010 | 0.009 | -0.011 |  | - | - | - |  |
| **PST** | **0.033** | 0.001 | -0.001 | 0.001 | 0.025 | **0.029** | **0.041** | -0.005 | 0.036 | 0.020 |  | - | - |  |
| **PWO** | 0.011 | 0.005 | -0.002 | -0.005 | 0.016 | 0.018 | 0.018 | 0.011 | 0.013 | 0.007 | 0.014 |  | - |  |
| **PWP** | 0.028 | 0.013 | 0.020 | 0.017 | 0.041 | 0.023 | 0.003 | -0.014 | 0.001 | 0.004 | 0.012 | 0.037 |  |  |


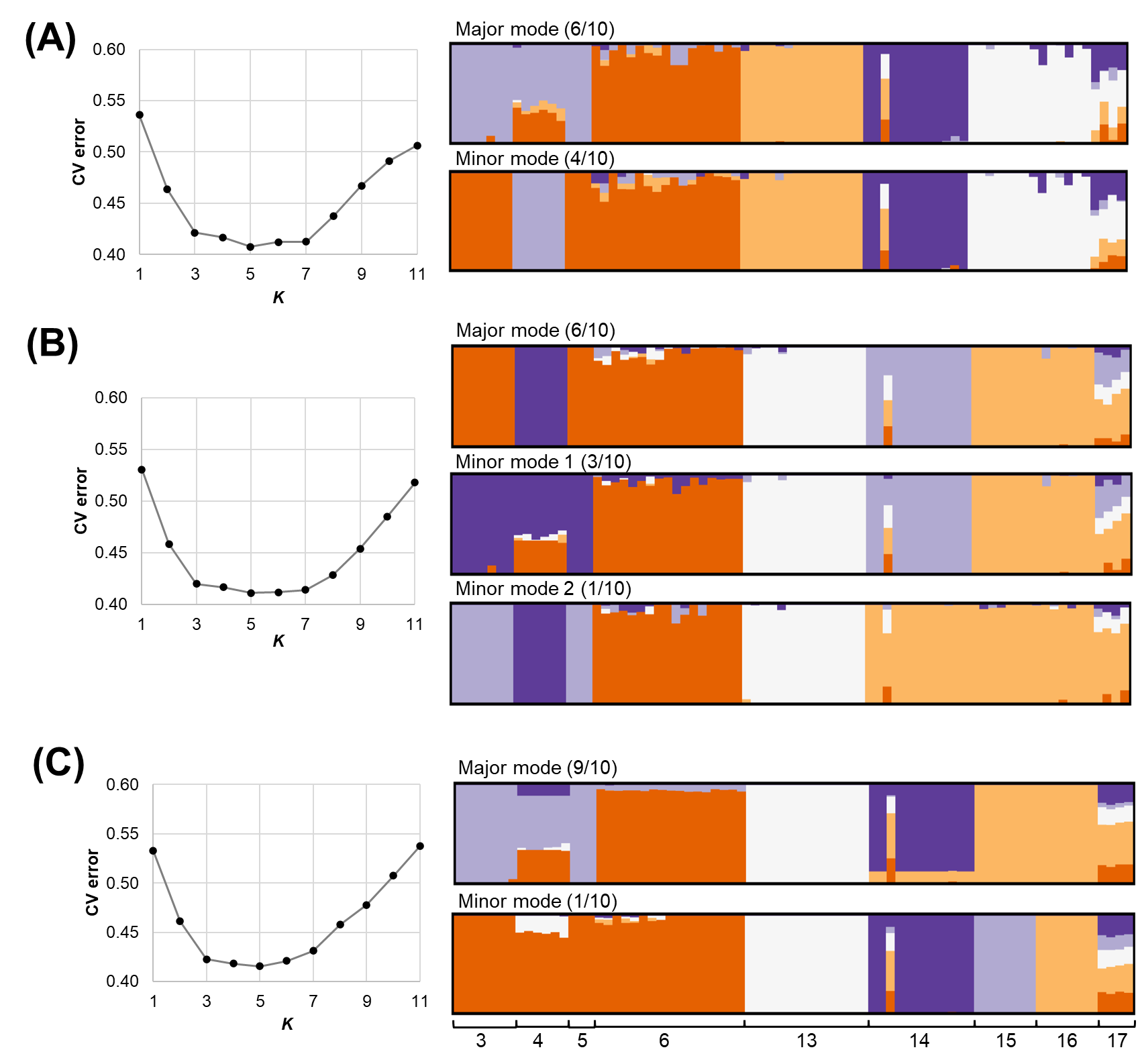


**Figure B1.** Visualization of population structure between neutral SNP datasets using contemporary tissue samples collected by Galbreath et al. (2009) compared for GT-seq panel design, composed of (A) 350 SNPs, (B) 700 SNPs, and (C) the full 15,480 SNP datasets. See Fig. 2 for geographic locations of sampling sites. Population structure was explored using the maximum likelihood individual assignment approach employed by ADMIXTURE (Alexander et al., 2009) for *K* = 5. Plots on the lefthand side indicate the optimal number of clusters (*K*) using the 5-fold cross validation (CV) error minimization approach by Alexander et al. (2009), for which the *K* that demonstrates the lowest CV error was selected as the optimal *K*. Figures on the righthand side show the estimated individual assignment of each sample (vertical bar) to one of five clusters (*K* = 5) for each sampled site, indicated on the x axis. The major and minor modes demonstrated when combining replicates using CLUMPAK (Kopelman et al., 2015) are shown, as well as the number of supporting replicates out of the ten conducted.


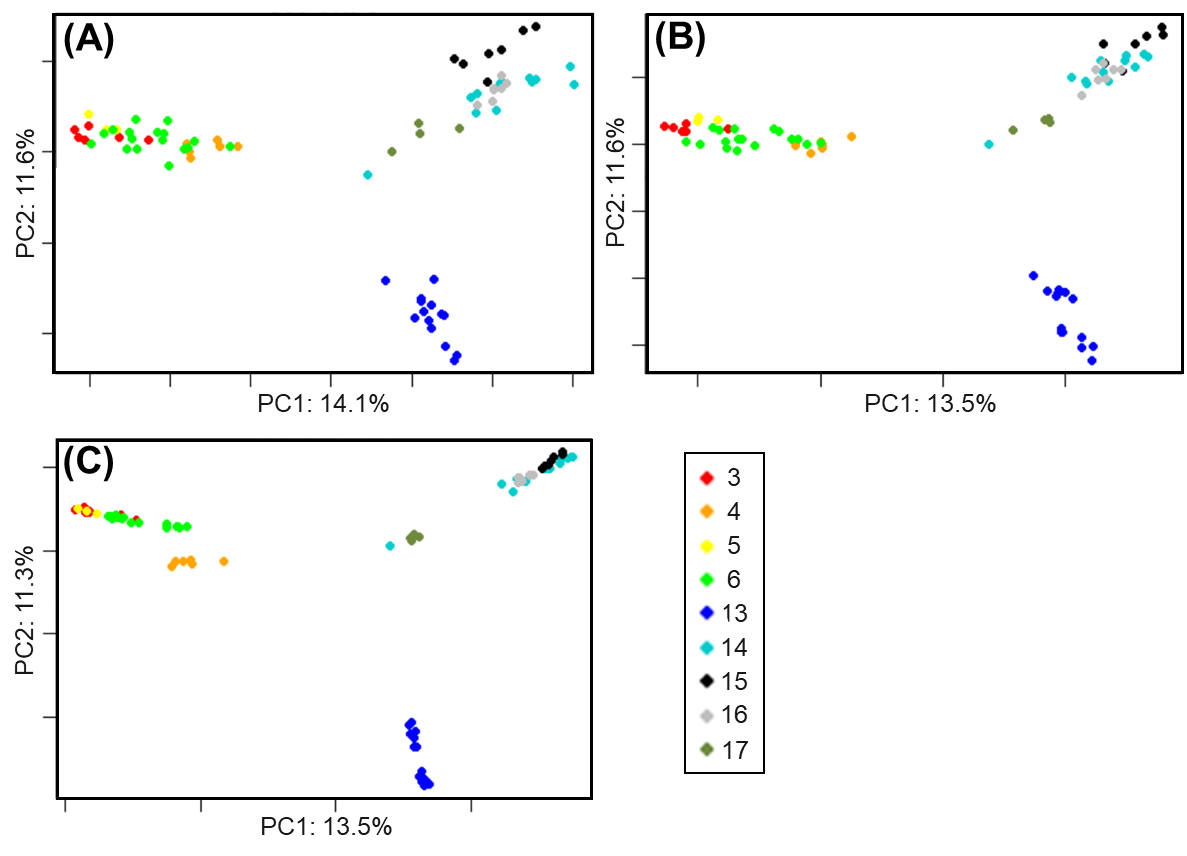


**Figure B2.** The principal component analysis (PCA) plots computed in the R package *smartsnp* (Herrando-Pérez et al., 2021) for (A) 350 SNPs, (B) 700 SNPs, and (C) the full 15,480 SNP datasets. See Fig. 2 for geographic locations of sampling sites.

**
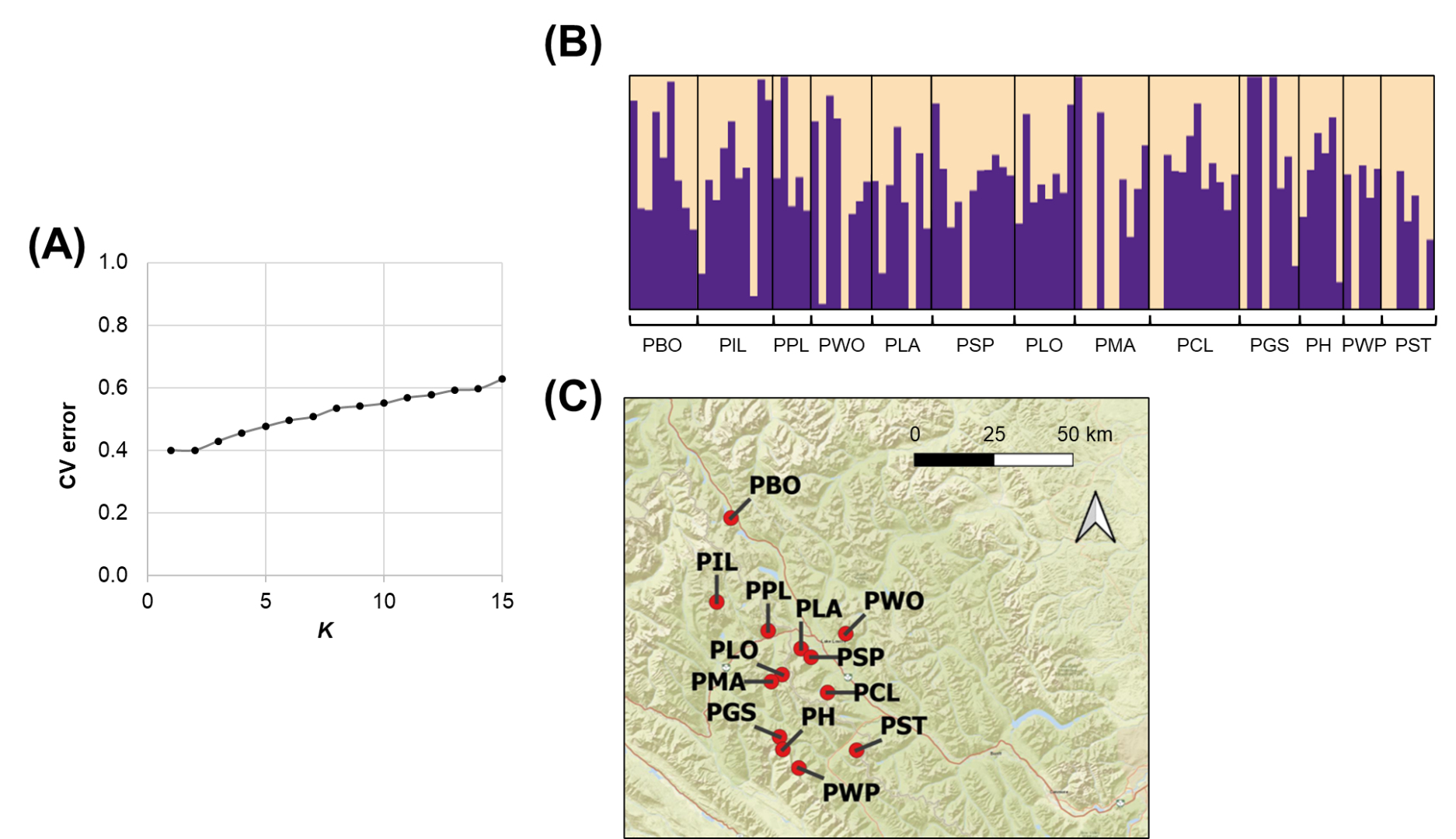
**

**Figure B3**. Reconstruction of population structure plots using the maximum likelihood approach implemented in ADMIXTURE 1.3.0 (Alexander et al., 2009) for fecal pellet samples (2021 and 2022 samples combined). Panel (A) shows the optimal number of clusters (*K*) using the 5-fold cross validation (CV) error minimization approach by Alexander et al. (2009), for which the *K* that demonstrates the lowest CV error was selected as the optimal *K.* Panel (B) shows the estimated individual assignment of each sample (vertical bar) to one of two clusters (*K* = 2) for each site. Sites are organized by relative geographic proximity, as shown in Panel (C) (created in QGIS® using data provided by © Esri).

**
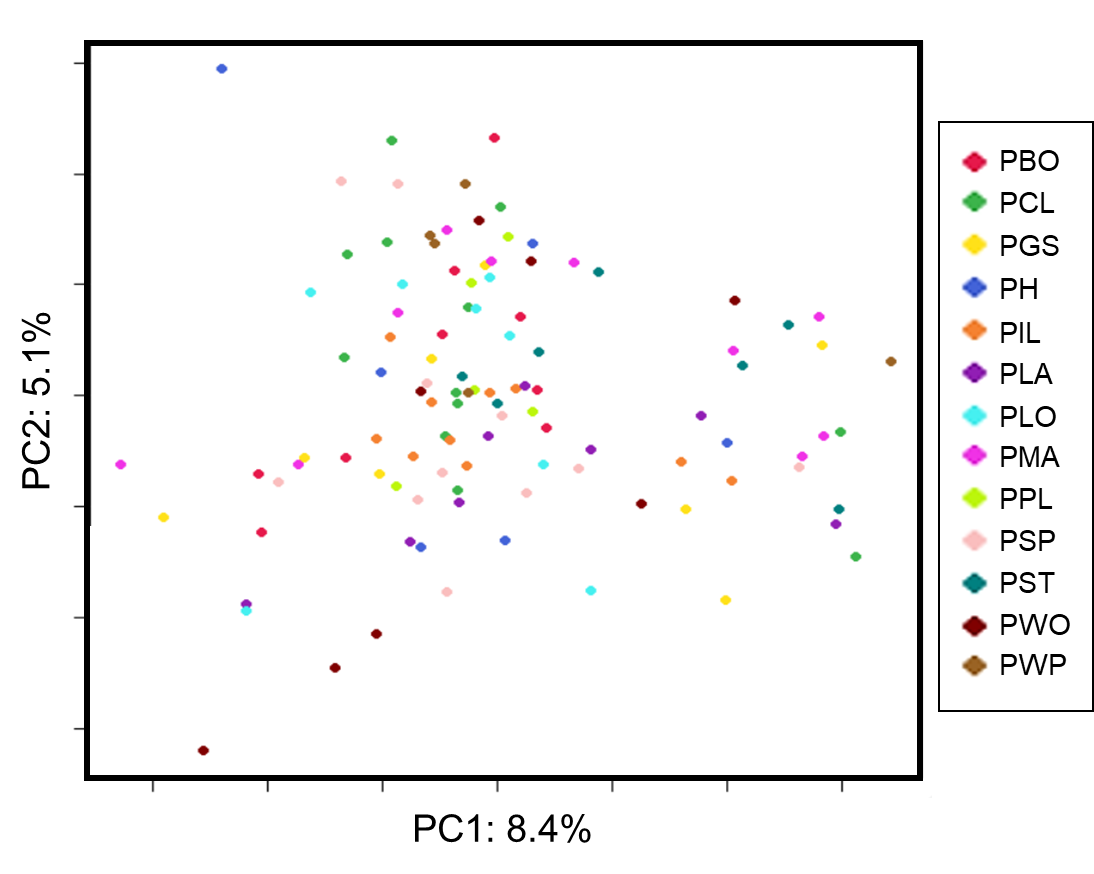
**

**Figure B4.** Principal component analysis (PCA) plots using the R package *smartsnp* (Herrando-Pérez et al., 2021) for fecal pellet samples among nine sites.
